# Scalable Generation of Pre‐Vascularized and Functional Human Beige Adipose Organoids

**DOI:** 10.1002/advs.202301499

**Published:** 2023-09-20

**Authors:** Mélanie Escudero, Laurence Vaysse, Gozde Eke, Marion Peyrou, Francesc Villarroya, Sophie Bonnel, Yannick Jeanson, Louisa Boyer, Christophe Vieu, Benoit Chaput, Xi Yao, Frédéric Deschaseaux, Mélissa Parny, Isabelle Raymond‐Letron, Christian Dani, Audrey Carrière, Laurent Malaquin, Louis Casteilla

**Affiliations:** ^1^ RESTORE Research Center Université de Toulouse, INSERM 1301, CNRS 5070, EFS, ENVT Toulouse 31100 France; ^2^ LAAS‐CNRS Université de Toulouse, CNRS, INSA Toulouse 31400 France; ^3^ CIBER “Fisiopatologia de la Obesidad y Nutrición”, Department of Biochemistry and Molecular Biomedicine University of Barcelona Madrid 28029 Spain; ^4^ Service de Chirurgie plastique, reconstructrice et esthétique Centre Hospitalier Universitaire Rangueil Toulouse 31400 France; ^5^ Faculté de Médecine Université Côte d'Azur INSERM, CNRS, iBV Nice 06103 France; ^6^ LabHPEC, Histology and Pathology Department Université de Toulouse, ENVT Toulouse 31076 France

**Keywords:** adipose‐derived stroma/stem cells (ASC), beige and brown adipocytes, guided‐assembly, hydrogels, microtissues, organoid morphogenesis, stromal vascular fraction

## Abstract

Obesity and type 2 diabetes are becoming a global sociobiomedical burden. Beige adipocytes are emerging as key inducible actors and putative relevant therapeutic targets for improving metabolic health. However, in vitro models of human beige adipose tissue are currently lacking and hinder research into this cell type and biotherapy development. Unlike traditional bottom‐up engineering approaches that aim to generate building blocks, here a scalable system is proposed to generate pre‐vascularized and functional human beige adipose tissue organoids using the human stromal vascular fraction of white adipose tissue as a source of adipose and endothelial progenitors. This engineered method uses a defined biomechanical and chemical environment using tumor growth factor β (TGFβ) pathway inhibition and specific gelatin methacryloyl (GelMA) embedding parameters to promote the self‐organization of spheroids in GelMA hydrogel, facilitating beige adipogenesis and vascularization. The resulting vascularized organoids display key features of native beige adipose tissue including inducible Uncoupling Protein‐1 (UCP1) expression, increased uncoupled mitochondrial respiration, and batokines secretion. The controlled assembly of spheroids allows to translate organoid morphogenesis to a macroscopic scale, generating vascularized centimeter‐scale beige adipose micro‐tissues. This approach represents a significant advancement in developing in vitro human beige adipose tissue models and facilitates broad applications ranging from basic research to biotherapies.

## Graphical abstract

This study reports a scalable tissue engineering strategy to generate vascularized human beige adipose tissue (AT) constructs. Organoids and macro‐scaled tissue demonstrate key functional features including cell plasticity associated with metabolic and paracrine activities adaptation specific to beige AT. This flexible system opens the way of multiple applications from basic science to biotherapies in the context of obesity and type‐2 diabetes.

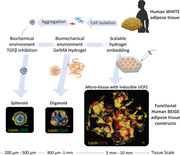

## 1 Introduction

Despite public health initiatives, type 2 diabetes and obesity have reached worldwide pandemic proportions and are associated with the leading cause of death. Both metabolic disorders are characterized by an imbalance between energy intake and energy expenditure. Adipose tissues are key regulators of this energy balance.^[^ ^1^ ^]^ While white adipose tissues (WAT) are the main energy storage of the organism, brown and beige adipose tissues are characterized by their inducible ability to dissipate energy upon activation, thanks to their specific uncoupling oxidative phosphorylation from ATP synthesis by the expression of the mitochondrial protein, uncoupling protein‐1 (UCP1).^[^ ^1^, ^2^ ^]^ Brown and beige adipose tissues also secrete the so‐called adipokines and batokines, supporting communication with surrounding cells as well as with distant organs.^[^ ^3^ ^]^

Contrary to brown adipose tissues, which are found in restricted depots, beige adipocytes may exhibit a white‐like phenotype and reside within specific WAT depots. These cells can be induced to display inducible thermogenic features through a reversible mechanism called “beiging”.^[^ ^4^, ^5^, ^6^ ^]^ In addition to cold exposure, several pathophysiological conditions activate beiging, including physical exercise^[^ ^7^ ^]^ and intermittent fasting.^[^ ^8^ ^]^ In adult humans, brown and beige adipocytes mass has been negatively correlated with obesity and aging.^[^ ^9^, ^10^ ^]^ Therefore, activation or mass increase of human beige adipose tissue have been investigated as therapeutic approaches to counteract metabolic disorders. As a consequence, developing models for investigating the emergence and maintenance of beige adipose tissue in humans has broad therapeutic interest for metabolic diseases including obesity and diabetes but also for aging. Such models would help to screen for drugs that can modulate intrinsic plasticity but also open the way for new biotherapy strategies. Indeed, the therapeutically beneficial effects of murine or human brown/beige adipocyte transplantation have been demonstrated in rodents where normoglycemia was recovered in diabetic mice and energy expenditure increased in obese mice.^[^ ^11^, ^12^, ^13^, ^14^ ^]^ However, translating this approach to patients requires developments in tissue engineering to allow the generation of human beige adipose tissue transplants that are clinically relevant in size and function. Recently, multiple approaches have been developed, including ours, to generate WAT models reproducing the 3D architecture and metabolic function of native tissues.^[^ ^15^, ^16^, ^17^, ^18^, ^19^, ^20^ ^]^ However, studies reporting relevant 3D beige adipose tissue models are scarcer, even more so with human cells. Approaches to engineering human beige adipose tissue largely rely on the differentiation of adipose progenitors in the absence of the vascular compartment^[^ ^21^, ^22^ ^]^ or the use of explant‐based culture^[^ ^23^ ^]^ that is prone to limited cell viability over time.^[^ ^24^ ^]^

When cultivated under suitable conditions, stem cells can undergo in vivo‐like morphogenesis and turn into structures containing a self‐organized cluster of cells called organoids. Such cultures possess the highest functional complexity and maturity obtained to date in vitro.^[^ ^25^ ^]^ However, growing organoids beyond the millimeter scale often leads to cell necrosis and/or incomplete maturation.^[^ ^26^ ^]^ These size limitations hinder recapitulation of the large‐scale features of tissue architecture and the development of organoid‐based therapeutic approaches. Recent tissue engineering approaches have been directed toward initiating and controlling cell autonomous modes of organogenesis at the microscopic and macroscopic scales while integrating a vascular system to sustain cell viability and function.^[^ ^27^ ^]^ Most of these approaches, use bottom‐up engineering strategies involving the generation of building blocks and subsequently their assembly.

In this process, critical parameters have been identified for driving multicellular responses toward in vitro tissue complexity.^[^ ^28^ ^]^ They include 1) controlling the cell types present to recapitulate tissue cell composition, 2) engineering chemically and mechanically permissive environments to promote intrinsic development programs, and 3) controlling the 3D patterning of cell clusters to define the shape and size of the final tissue construct. In pursuit of these aims, engineered biomaterials, such as hydrogels, are promising tools to promote the spatiotemporal growth of organoids and shape‐guided morphogenesis.^[^ ^28^ ^]^ Their porous structure provides both physical support for cell adhesion, proliferation, and migration as well as molecular diffusion properties close to the native extracellular matrix.^[^ ^29^ ^]^ Hydrogels open the way toward robust, scalable processes for 3D structuration using bioprinting technology.^[^ ^30^ ^]^ Photopolymerizable gelatin methacryloyl hydrogels (GelMA) have been increasingly used for tissue engineering applications to define microenvironments that are hard to achieve with other naturally derived hydrogels such as Matrigel.^[^ ^29^, ^31^, ^32^ ^]^

**Figure 1:**
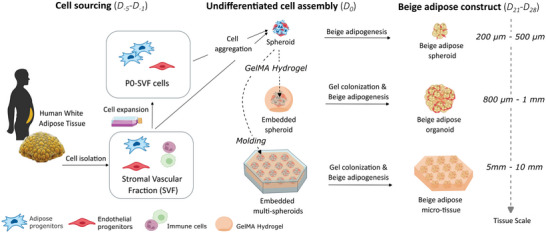
Multiscale approaches to generate vascularized human beige adipose tissue constructs. SVF cells purified from human white adipose tissue were used directly or after amplification in 2D culture to generate spheroids. Spheroids can be individually embedded or multiply assembled using a GelMA mold. A specific combination of volume per spheroid, percentage, and reticulation of GelMA embedding condition in an optimized cocktail medium leads to the generation of functional human beige organoids as well as beige micro‐tissue.

Here we develop a straightforward, multiscale approach promoting the 3D self‐organization of spheroids into pre‐vascularized human beige adipose organoids. Contrary to classic bottom‐up engineering approaches which aim to generate building blocks followed by their ultimate assembly,^[^ ^33^, ^34^ ^]^ we developed a scalable guided‐assembly strategy of spheroids. The system is based on GelMA hydrogel and stromal vascular fraction (SVF) cells from human WAT as a source of endothelial progenitors and multipotent adipose‐derived mesenchymal stem cells (ASC). We defined a controlled biochemical and mechanical environment allowing ASC commitment toward the beige adipocyte lineage while ensuring vascular development at the microscopic scale through tumor growth factor β (TGFβ) pathway inhibition and tuning of GelMA porosity and mechanical properties by varying gelatin percentages to embed individual spheroids. The functionality of the resulting micro‐scaled construct and its translation to a multi‐spheroid macro‐scaled tissue construct was then demonstrated (**Figure** **1**).

## 2 Experimental Section

### 2.1 Hydrogel Preparation

#### 2.1.1 GelMA Synthesis

A solution of porcine skin gelatin type A (110 bloom, Sigma, USA) was prepared in carbonate‐bicarbonate (CB, 0.25 m) buffer (0.075 mol Na2CO3 and 0.175 mol NaHCO3 in 1 L of dH2O, adjusted to pH 9 using 5 m NaOH or 6 m HCl). Gelatin (20%, w/v) was dissolved in CB buffer (60 C for 1–2 h), the solution cooled to 50 °C and methacrylic anhydride (Sigma, USA) was added with magnetic stirring (3 h at 50 °C; methacrylic anhydride/gelatin feeding ratio: 0.1 mL g^−1^). The pH was readjusted to 7.4 to terminate the reaction and the solution was filtered and dialyzed (MW CO 10 000) against distilled water (3 days at 40 °C) to remove excess methacrylic acid and salts. Dialysate was changed every 12 h. This solution was lyophilized for 3 days and stored at 4 °C until further use.^[^ ^35^ ^]^ 1H‐proton magnetic resonance spectroscopy (Bruker) quantified methacyloyl substitution to be 63%.^[^ ^36^ ^]^

To prepare GelMA solution, lyophilized GelMA was dissolved in D‐PBS (5, 8, 10, or 15% w/v) with photoinitiator lithium phenyl‐2,4,6‐trimethyl‐benzoylphosphinate (LAP, 0.1% w/v, Sigma, USA). GelMA solution was kept at 4 °C until use.

#### 2.1.2 GelMA Rheological Characterization

A mechanical tester (Mark‐10 ESM, USA) was used to test the storage modulus of GelMA hydrogels. The system was operated with a 5 N load cell at a displacement speed of 5 mm min^−1^ speed with a 20% deformation threshold. GelMA solutions prepared as described above were introduced in cylindrical molds (6 mm in diameter × 4 mm in thickness) and crosslinked by exposing them to 405 nm light (Formlabs, USA) at room temperature (RT) for various durations (10 s–3 min). The samples were kept in PBS for 8 h before testing. Conservation moduli of hydrogels were calculated from the slope of the very first linear region of the stress–strain curve. The evolution of the mechanical properties was also investigated by performing mechanical tests for 7 days (immersion in PBS at 37 °C) after preparation (*n* = 5).

#### 2.1.3 Electron Microscopy

Hydrogel samples were frozen in liquid nitrogen, lyophilized for 6 h and sputter coated with Au (10 nm). Morphology was analyzed with a scanning electron microscope (Hitachi S‐4800S‐4800, Japan). Dimensional analysis of porosity and pore sizes was performed with Image J software (NIH).

### 2.2 Preparation of Anti‐Adhesive PDMS Surface and Molds for GelMA Molding

Polydimethylsiloxane (PDMS) molds were prepared by casting on 3D‐printed templates. 3D templates were obtained by stereolithography using a DWS 29J+ system (DWS, Italy) and DL260 photoresist (DWS, Italy). Once fabricated, the 3D templates were treated using perfluorodecyltrochlorosilane (FDTS) after SiO2 coating using the SPD system. PDMS (Sylgard 184, Dow Corning) was prepared in a 10:1 (Base:Curing agent) ratio, degassed under vacuum, and poured into the 3D‐printed mold. Crosslinking of PDMS was performed for 2 h at 60 °C. PDMS molds were then removed manually from the templates. PDMS molds were then incubated overnight at RT with an anti‐adhesive treatment using pluronic F127 (20 mg mL^−1^, Sigma, USA) followed by three washes in D‐PBS and air‐drying before use. For GelMA droplet formation, a flat pluronic‐coated PDMS surface was prepared similarly by pouring PDMS into a petri dish. After curing, pluronic treatment was performed without further removal of PDMS.

### 2.3 Generation of Beige Adipose Tissue Spheroids and Culture

#### 2.3.1 Isolation and Amplification of SVF Cells from Human Adipose Tissue

Human SVF was isolated from abdominal dermolipectomy (plastic surgery department, CHU Toulouse, France) of female donors (body mass index ranging from 22.3 to 27.9 kg m^−2^). The experimental protocols were approved by the French research ministry's institutional ethics committee (No: DC‐2015‐23‐49) and informed consent was obtained from all subjects in accordance with institutional guidelines on human tissue handling and use. Adipose tissue was mechanically dissociated and enzymatically digested for 45 min at 37 °C using collagenase NB4 (13.6 U mL^−1^, Coger, Germany) in α‐Minimum Essential Medium (Life‐Technologies, UK), supplemented with amphotericin B (0.1% v/v, Life‐Technologies, UK), and streptomycin/penicillin (1% v/v, Life‐Technologies, UK) hereafter named αMEM‐ASP. After filtration on a 100 µm nylon net filter (Steriflip, Millipore, USA) and centrifugation (600 g, 10 min), cells were washed in αMEM‐ASP and centrifuged again (600 g, 5 min). The cell pellet was resuspended in erythrocyte lysis buffer (eBioscience RBC Lysis Buffer Multi‐species, Life‐Technologies, UK) and incubated for 5 min at room temperature (RT). Isolated SVF cells were then centrifuged (600 g, 5 min) and resuspended in Endothelial Growth Medium‐2 (EGM2, PromoCell, Germany) supplemented with ASP. Cells were seeded directly in suspension for SVF spheroid formation or at 4000 cells cm^−2^ in 2D culture for further amplification. 2D cultures were maintained in EGM2 until they reached 80% confluency and the medium was changed every 3 days. The resulting amplified cells (P0‐SVF) were used for spheroid formation or adipocyte differentiation in 2D cultures.

#### 2.3.2 Spheroid Formation

Spheroids were formed from either SVF or P0‐SVF cells. To promote cell aggregation, 50 000 cells were seeded in a reduced volume of EGM2 medium (50 µL) in ultra‐low attachment (ULA) 96‐well round‐bottom plates (Corning Incorporated Life Sciences, USA) and maintained overnight under stirring (150 rpm). For SVF cells, to further improve cell aggregation, cell seeding was followed by plate centrifugation (600 g for 5 min). The following day, EGM2 (150 µL) was added to each well. Cells were maintained in the proliferation medium until spheroid formation, i.e., 5 days for SVF‐spheroids and 1 day for P0‐SVF‐spheroids.

#### 2.3.3 Individual Spheroid Embedding

To generate GelMA embedded spheroids, once formed, spheroids were mixed with pre‐warmed GelMA/0.1% LAP solution (37 °C, 10 min). Spheroids were then individually pipetted in a defined volume of GelMA/0.1% LAP solution (1.5 µL, 3 µL) and dispensed onto an anti‐adhesive PDMS surface, prepared as described above. GelMA droplets containing one spheroid were then photo‐crosslinked via exposure to 405 nm light for 40 s (Form cure, Formlabs, Germany). It has to be noted that for GelMA 5%, crosslinking duration was increased to 60 s as lower durations did not permit the generation of droplets solid enough for manipulation. Embedded spheroids were individually transferred into 24‐well flat‐bottom ULA plates (Corning Incorporated, Lifes Sciences, USA) and maintained in an EGM2 proliferation medium for 7 days before differentiation. Half of the medium was changed every 2–3 days.

#### 2.3.4 Multi‐Spheroid Construct Generation

Multi‐spheroid constructs were prepared by casting GelMA and positioning spheroids on a PDMS mold template with spherical cavities (Diameter: 1400 µm, height: 700 µm). Spheroids were handled using a manual aspiration method and positioned, thanks to a digital camera, in the middle of the mold microwell filled with GelMA. When all the spheroids (14 spheroids per mold) were precisely positioned, GelMA was cured for 40 s with 405 nm light (Form cure, Formlabs, Germany). The GelMA multi‐spheroid construct was unmolded before the culture process.

#### 2.3.5 Adipocyte Cell Differentiation in 2D and 3D Cultures

Differentiation onset varied according to the type of culture. For 2D culture, P0‐SVF cells were first seeded at 80 000 cells cm^−2^ on gelatin (0.1%, Sigma, USA) coated plates in EGM2 medium. Differentiation was then initiated when cells reached confluency. For spheroids in the absence of GelMA hydrogel, differentiation was initiated once spheroids were formed. For GelMA embedded spheroids, either individually or in multiple constructs, differentiation was initiated after a proliferation phase of 7 days in EGM2 medium. For all types of cultures, cells were differentiated for 21 days with appropriate adipogenic cocktails. Half of the medium was changed every 3–4 days. Cells were differentiated using variations of an adipogenic cocktail previously described by the team.^[^ ^18^ ^]^ These adipogenic cocktails consist of αMEM‐ASP supplemented with fetal bovine serum (FBS) (2%), insulin (5 5 µg mL^−1^), apotransferin (10 µg mL^−1^, Sigma, USA), bone morphogenetic protein 7 (BMP7, 50 ng mL^−1^, MiltenyiBiotec, France) with or without intralipids (0.2% diluted from 20% emulsion, Sigma, USA). When specified, the TGFβ pathway inhibitor SB431542 (MiltenyiBiotec, Germany), also referred to as SB4, was added to the adipogenic cocktail (5 µm). For treatment with UCP1 inducers, cells were treated 3 days prior to the end of the differentiation process with rosiglitazone (100 nm, Sigma, USA), 3,3′’,5‐triiodo‐L‐thyronine (T3, 0.2 nm, Sigma, USA), all‐trans retinoic acid protected from light (0.1 µm, Sigma, USA), 8‐(4‐chlorophenylthio) ‐adenosine 3′',5′'‐cyclic monophosphate (8‐CPT‐cAMP, 200 µm, Abcam, UK). All‐trans retinoic acid treatment was renewed every day until the end of the culture to overcome its molecular instability.

### 2.4 Cell Viability Assay

#### 2.4.1 Spheroid Size Measurements

Imaging of spheroid size was performed during the culture process at indicated times using a Nikon eclipse TE2000‐5 microscope with a 10X objective. Spheroid area was measured using Fiji software (National Institutes of Health, USA). Six to eight spheroids were measured for each time point per human sample. Nine human samples were analyzed.

#### 2.4.2 Propidium Iodide Staining

3D image‐based cell viability quantification was conducted by staining free spheroids and embedded spheroids with propidium iodide (10 µg mL^−1^, Invitrogen, USA) in a culture medium for 1 h at 37 °C. After three D‐PBS washes, samples were fixed with 4% paraformaldehyde. The fixed cultures were permeabilized and stained with DAPI as described above. Samples were washed three times with D‐PBS (30 min, RT) and cleared for at least 48 h with Scale S4 solution^[^ ^37^ ^]^ before imaging. All samples were imaged using a confocal microscope (LSM 880, Carl Zeiss, France). The total number of nuclei and IP+ nuclei were quantified using ImageJ. Prior to nuclei counting, the DAPI signal was segmented using two‐dimensional (2D) Stardist plugging,^[^ ^38^ ^]^ a deep‐learning‐based method for 2D nucleus detection. The mean of three spheroids or GelMA‐embedded spheroids was calculated for each condition per human sample. For quantification on embedded multi‐spheroid constructs, the mean of two peripheral and two central spheroids was calculated to take into account a putative heterogeneity. The average viability percentage was calculated as the number of propidium iodide + nuclei/DAPI+ nuclei per slice.

### 2.5 DNA Quantification

DNA quantification was performed to assess cell proliferation and maintenance. DNA was extracted from an average of 24 spheroids or five GelMA‐embedded spheroids according to the blood and tissue DNA extraction kit (Qiagen) manual. Spheroids were washed with D‐PBS and lysed ALT/proteinase K buffer (200 µL) overnight under mixing (800 rpm). To ensure DNA purity, RNase A (Qiagen) was added to the samples and incubated for 2 min at RT before the addition of a 1:1 AL/100% ethanol mix. DNA was detected with the 1x QubitTM High sensitivity dsDNA kit according to the manufacturer's instructions. Fluorescence intensities were measured with Qubit 4.0 fluorometer (Invitrogen, CRCT, Toulouse). Data were expressed as DNA quantity/spheroids (ng).

### 2.6 RNA Extraction and Quantitative Relative Real‐Time PCR

Cell samples were homogenized in QIAzol lysis reagent (Qiagen, USA). 3D culture samples were further disrupted for 2 min at 30 Hz using Tissue Lyser (Qiagen). To avoid extraction bias, a pool of 14 embedded single spheroids was used to compare with the expression of one embedded multi‐spheroid made of 14 spheroids. Total RNA was isolated using phenol‐chloroform extractions followed by Quick‐RNA microprep kit procedure (Zymo Research, USA) and reverse transcribed into cDNA using high capacity cDNA reverse transcription kit (Applied Biosystems, USA). qPCR was performed using a StepOne system (Applied Biosystems, USA) with Fast SYBR Green Master Mix supplemented with 1/10e diluted cDNA and primers (300 nm) listed in Table S1 (Supporting Information). Relative gene expression was calculated by the 2−ΔΔ*CT* method. The Δ*Ct* was obtained by normalizing mean expression values of each gene to the geometric mean of the reference genes, ribosomal protein lateral stalk subunit P0 (RPLP0), glucuronidase beta (GUSB), peptidylprolyl isomerase A (PPIA), and tyrosine 3‐monooxygenase/tryptophan 5‐monooxygenase activation protein zeta (YWAZ). The ΔΔ*Ct* was calculated by normalizing conditions to 2D undifferentiated cells for 2D experiments or to non‐embedded undifferentiated spheroids for 3D culture experiments.

### 2.7 Immunofluorescence Analysis

2D and 3D cultures were fixed with 4% paraformaldehyde at RT. In the case of 3D culture, after D‐PBS washing, samples were permeabilized and blocked in D‐PBS solution supplemented with Triton X‐100 (1%, Sigma, USA) and horse serum (3%, Jackson Immunoresearch, UK) for 3 h at RT. Samples were then incubated with the primary antibody in D‐PBS solution supplemented with horse serum (1%) and Triton X‐100 (1%), at the appropriate dilution (Table S2, Supporting Information), overnight at RT. After three washes in D‐PBS, secondary antibodies coupled to Alexa‐488, Alexa‐ 594 or Alexa‐647, (1:500, Life Technologies, UK) in D‐PBS supplemented with horse serum (1%) and Triton X‐100 (1%) were added as specified, 3 h at RT. For lipid droplet staining, 493‐Bodipy (2 µg mL^−1^, Life Technologies, UK) was added to the solution. After D‐PBS washes, nuclei were stained with DAPI (2 µg mL^−1^), 1 h at RT (Sigma, USA). For 2D culture, the same protocol was used but incubation durations were shortened for the different steps. For 3D culture imaging, samples were cleared for at least 48 h in Scale S4 solution^[^ ^39^ ^]^ composed of (w/v) D‐(‐)‐sorbitol (40%, Sigma, USA), glycerol (10% w/v, Euromedex, France), urea (4 m, Sigma, USA), Triton X‐100 (0.2%), dimethylsulfoxide (20% v/v, Sigma, USA). Samples were analyzed by confocal imaging (LSM 880, Carl Zeiss, France) and images were processed using Fiji software (National Institutes of Health, USA).

### 2.8 ProteinSimple Capillary Immunoassay

For western analysis, 24 spheroids or 12 embedded spheroids were washed with PBS 1x and resuspended in ice‐cold RIPA buffer (100 µL, Sigma, R0278, USA) adjusted with SDS (2%) and completed with anti‐protease and phosphatase inhibitors. For protein extraction from mouse tissues, the volume of the complete RIPA buffer was adjusted (1 mL per 100 mg). Samples were mechanically dissociated with Precellys tissue homogenizer at 4 °C. Protein lysates were then transferred into other tubes and sonicated with an ultrasonic homogenizer twice for 30 s in ice at 20 kHz. Samples were centrifuged for 10 min at 10 000 rpm and whole protein cell extracts were quantified by the Lowry method. Samples were run with proteinSimple capillary electrophoresis immunoassay according to the ProteinSimple user manual. Briefly, protein extracts (1.5 ng well^−1^) were mixed with dithiothreitol (40 mm, DTT) and master mix (ProteinSimple). Samples were heated at 95 °C for 5 min and dispensed into a designated plate along with blocking reagent, primary antibodies Horseradish peroxidase (HRP)‐conjugated secondary antibodies, chemiluminescent substrate, and total protein detection reagents. All electrophoresis and immunodetection steps were automatized within the capillary system (ProteinSimple Jess). Chemiluminescence intensities were quantified with Compass software (ProteinSimple) and normalized to the total protein signal.

### 2.9 Lipolysis Assay

A lipolysis assay measuring glycerol release was conducted on embedded spheroids after differentiation using Free Glycerol Reagent kit (Sigma, USA) according to the manufacturer's recommendations. At the end of the differentiation, protocol‐embedded spheroids were transferred into 96 wells of ULA plate in phenol red‐free DMEM medium (90 µL) supplemented with glucose (5 mm). After 24 h of lipolysis stimulation with 8 cpt‐AMPc (200 µm), media (20 µL) was collected and added to the reaction mix. After 15 min of incubation, absorbance was read at OD 540 nm, and glycerol released was calculated using a standard curve. Four days prior to the experiment, the adipogenic medium was depleted of insulin to prevent lipolysis inhibition. Data are normalized by DNA quantity.

### 2.10 Metabolic Functionality

#### 2.10.1 Lactate and Glucose Measurements

Extracellular levels of lactate and glucose were measured after treatment with UCP1 inducers to assess the change in metabolic activity of embedded spheroids. Lactate and glucose levels were measured with the Lactate Pro II test meter (Arkray) and Contour XT TS (Bayer), respectively. To account for increases in molecular concentration that could arise from medium evaporation, differences in glucose and lactate levels were calculated in comparison to evaporation in control wells without cells. Data are normalized to DNA quantity.

#### 2.10.2 Seahorse Metabolic Assay

Metabolic profiling of embedded spheroids was performed by evaluating the oxygen consumption rate (OCR) of cells using the Seahorse XF24 Extracellular Flux Analyzer (Seahorse Biosciences) in the XF24 islet capture plate. Two embedded spheroids were placed in a well of an islet capture microplate (Agilent). Once in position, the culture medium was replaced with assay XF Seahorse DMEM medium (500 µL) supplemented with glutamine (2 mm), glucose (10 mm), and pyruvate (1 mm). Embedded spheroids were incubated for 45 min in a CO_2_‐free incubator at 37 °C prior to metabolic analysis. During Seahorse XF cell Mito stress run, cells were first exposed to 8cpt‐AMP (200 µm) to assess for adrenergic stimulation. Sequential injections of inhibitors of key components of cellular respiration were used to determine metabolic parameters such as basal, maximal, or uncoupled respiration. First, oligomycin (3 µm) was added to inhibit ATP synthase and reveal uncoupled respiration. Then, uncoupler FCCP (3 µm) was added to induce maximal respiration. Finally, a mix of rotenone (3 µm) and antimycin A (3 µm) that inhibit complexes I and III in the electron transport chain was added to determine non‐mitochondrial respiration. Basal and maximal respirations were calculated by subtracting non‐mitochondrial respiration from OCR obtained before oligomycin injection and after FCCP injection, respectively. All values were expressed as a percentage of maximal respiration. Five human samples were analyzed. For each condition, five wells were used to measure mean OCR values from each human donor.

### 2.11 Measurement of Organoid Protein‐Secreted Factors

Levels of secreted interleukin‐6 (IL6), meterorin‐like, growth/differentiation facto‐r‐15 (GDF15), and chemokine C‐X‐C ligand‐14 (CXCL14) were measured using ELISA. Medium was collected at the end of the culture process and stored at −80 °C. IL6, meterorin‐like, GDF15 and CXCL14 levels were determined using commercial ELISA kits (R & D Systems, Minneapolis, USA, # D6050, # DY7867‐05, # DY957; Ray Biotech, Peachtree Corners, USA, # ELH‐CXCL14, respectively). Medium that had not been in contact with cells was used as a detection control for ELISA assays. Data are normalized to DNA quantity.

### 2.12 Statistical Analysis

All results are presented as mean values of independent experiments, each from a different donor, ± standard deviation. The normal distribution of data was tested using the Kolmogorow–Smirnow test and homoscedasticity with Levene test. Significant differences among groups were evaluated using parametric two‐sample *t*‐test students or one‐way analysis of variance (ANOVA‐1) followed by post hoc analysis with Tukey's multiple comparison test unless stated otherwise. Significant fold differences compared to a reference control were analyzed by one‐sample *t*‐test. For qPCR analysis, statistical tests were performed on log2 fold change (−ΔΔ*Ct* values).^[^ ^40^ ^]^ *p*‐values ≤ 0.05 were considered significant.

## 3 Results

### 3.1 TGFβ Inhibition Promotes Beige Adipogenesis and Vascular Network in 3D Spheroid Model using P0‐SVF

**Figure 2:**
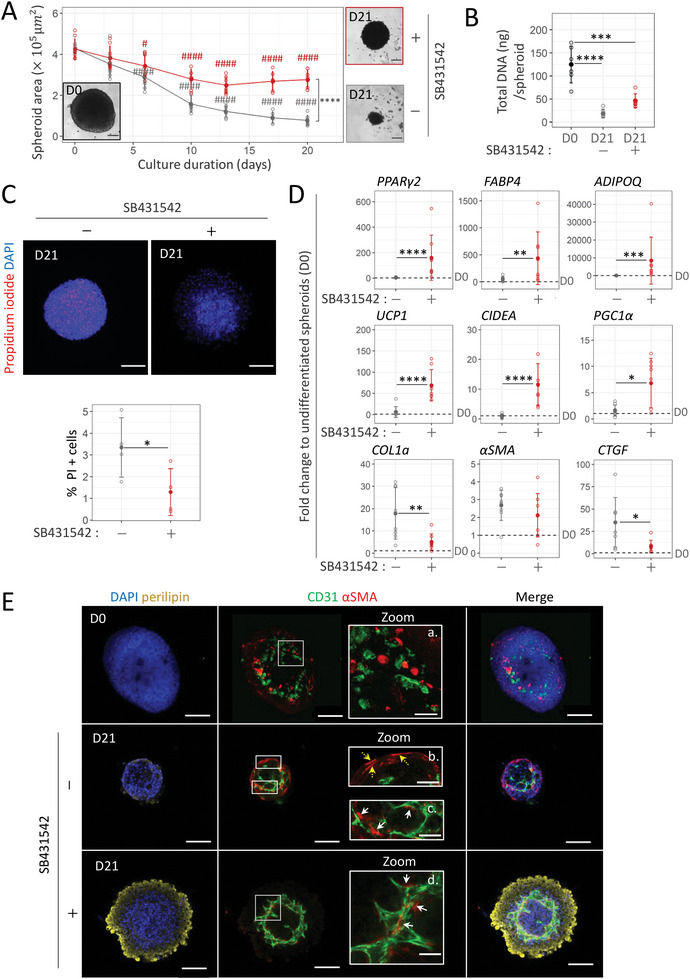
TGFβ inhibition promotes adipocyte differentiation and vascular formation while preventing the shrinking of 3D spheroids. A). Changes in spheroid size with differentiation duration in adipogenic medium in the absence (bottom curve, in gray) or presence (top curve, in red) of SB431542 (5 µm), an inhibitor of TGFβ pathway (*n* = 9). Brightfield images of spheroids before and after differentiation in each media. Statistical analysis was performed by two‐way ANOVA followed by post‐hoc Tukey's multiple comparisons (#: D0 versus Dx time point for each culture media, *: − vs + SB431542). B) Average DNA content per spheroid before (D0) and after differentiation (D21) in adipogenic medium with (in red) or without SB431542 (in gray) (*n* = 6). C) Confocal imaging and quantification of propidium iodide (dead cells in red) staining with respect to DAPI (blue) staining within P0‐SVF spheroids after differentiation with (in red) or without SB431542 (in gray) (*n* = 4). D) Gene expression analysis of beige adipocyte markers (*UCP1*, *CIDEA*, and *PGC1α)*, generic adipocyte markers (*PPARγ2*, *FABP4*, and *ADIPOQ*), and myofibroblast markers (*αSMA*, *COL1a*, and *CTGF*) in spheroids after 21 days of differentiation in the presence or absence of SB431542 (*n* = 8). Data are expressed relative to undifferentiated spheroids. E) Immunofluorescence analysis within P0‐SVF spheroids at days 0 and 21 of differentiation under each media condition. Specific antibodies against human lipids containing cells marker perilipin (yellow), endothelial cell marker CD31 (green), and α‐smooth muscle actin (SMA) (red) were used. DAPI staining highlights cell nuclei. Immunofluorescence images of lipids‐containing cells, endothelial cells, and pericytes revealed by perilipin (yellow), CD31 (green), and αSMA (red) staining, respectively, within P0‐SVF spheroids at day 0 and 21 of differentiation under each media condition. DAPI staining highlights cell nuclei. Scale bar: 200 µm. White squares (a–d) show areas enlarged to highlight the organization of endothelial cells and αSMA+ cells inside spheroids. Scale bar: 50 µm. Under both conditions, αSMA+ cells that aligned with CD31+ cells could be observed (white arrows). In the absence of SB431542 in adipogenic medium, peripheral αSMA+ cells can also be observed (yellow arrows) independently of CD31+ cells. All quantitative values are shown as mean ± standard deviation. Statistical analyses were performed by two‐sample *t*‐test or two ways ANOVA. Statistical significances: ^*^ *p* ≤ 0.05, ^**^ *p* < 0.01, ^***^ *p* < 0.001, and ^****^ *p* < 0.0001.

Within adipose tissue, adipocytes reside in close proximity to blood vessels which are known to have a key role in beige adipose tissue physiology. Thus, it is very important to preserve the two cell compartments for the generation of a physiologically relevant adipose tissue model. To promote commitment to beige adipose tissue while preserving endothelial cell development in a 3D context, we first optimized a differentiation medium based on an adipogenic cocktail previously developed by our team.^[^ ^18^ ^]^ In particular, the removal of intralipids known to promote hypertrophy of white adipocytes^[^ ^41^ ^]^ was investigated. Although the lower adiponectin (*ADIPOQ*) mRNA levels and lipid content showed an overall reduction of adipocyte differentiation, *UCP1* expression increased significantly when intralipid levels were removed in 2D culture conditions. This was associated with increased cluster of differentiation 31 (CD31) expression and the development of branched CD31+ cell networks (Figure S1A,B, Supporting Information). This beige adipogenic medium was then tested on 3D spheroids generated from amplified SVF cells (P0‐SVF cells). Spheroid formation was associated with the loss of a certain number of cells mostly located in the inner part of the spheroid (2.84% +/− 0.66% positive cells by propidium iodide staining, data not shown). Once in culture, spheroid size significantly decreased along differentiation (**Figure** **2**A, in gray), associated with reduced DNA content (Figure 2B, in gray). 3% of dead cells were also observed by day 21, as revealed by propidium iodide staining (Figure 2C, in gray). In contrast to 2D culture, the adipogenic medium was not sufficient to induce adipocyte differentiation of P0‐SVF cells when cultivated as spheroids. Indeed, levels of adipocyte‐specific mRNAs were comparable to undifferentiated cells (D0) (Figure 2D, in gray), and perilipin staining revealed a lack of this lipid droplet‐associated protein (Figure 2E, middle panel).

Regarding endothelial cell development, while CD31 positive and α smooth muscle actin (αSMA) positive cells were disorganized at day 0 (D0, zoom a. and D21, zoom c., Figure 2E) this medium enabled the self‐organization of CD31+ endothelial cells into pseudo‐vascular networks in spheroids. These CD31+ cells aligned with αSMA‐expressing cells akin to mural cells. However, we also observed αSMA+ cells independent of CD31+ endothelial cells at the periphery of the spheroid (D21, zoom b, Figure 2E). Taken together with the upregulation of collagen 1 (*COL1a*) and connective tissue growth factor (*CTGF*) mRNA levels compared to undifferentiated spheroids (Figure 2D, in gray), these data suggest induction of myofibroblast differentiation.

Since the TGFβ pathway favors ASC differentiation into myofibroblasts at the expense of adipogenesis,^[^ ^42^ ^]^ spheroids were treated with an inhibitor of the TGFβ pathway, SB431542, throughout the differentiation process. TGFβ pathway inhibition significantly reduced spheroid shrinking during differentiation (Figure 2A, in red), loss of DNA content (Figure 2B, in red), and cell death (Figure 2C, in red). Interestingly, in P0‐SVF spheroids, TGFβ pathway inhibition led to a significant increase in gene expression of adipogenesis markers, including increased beige‐specific transcripts (*UCP1*, *CIDEA*, and *PGC1α*). This increase was also associated with a decrease in the expression of myofibroblast markers *CTGF* and *COL1a* (Figure 2D, in red). Immunofluorescence experiments also revealed that TGFβ inhibition did promote the appearance of perilipin‐expressing cells, which were mainly located at the periphery of the spheroid (Figure 2E, lower panel). Notably, TGFβ inhibition decreased the presence of αSMA+ cells at the periphery of the spheroids but did not interfere with the development of αSMA+ cells found in the vicinity of CD31+ endothelial cells inside the spheroid (Figure 2E, zoom d). Although TGFβ pathway inhibition limited spheroid shrinking, it was not sufficient to completely avoid spheroid size reduction and loss of DNA content during differentiation. We hypothesized that providing spheroids with an appropriate biomechanical environment through hydrogel embedding might further help to generate tissue constructs containing an increased number of viable cells while further increasing beige adipocyte differentiation and vascular formation.

### 3.2 Adequate GelMA Embedding of Spheroid Supports Cell Mass Expansion using P0‐SVF

**Figure 3:**
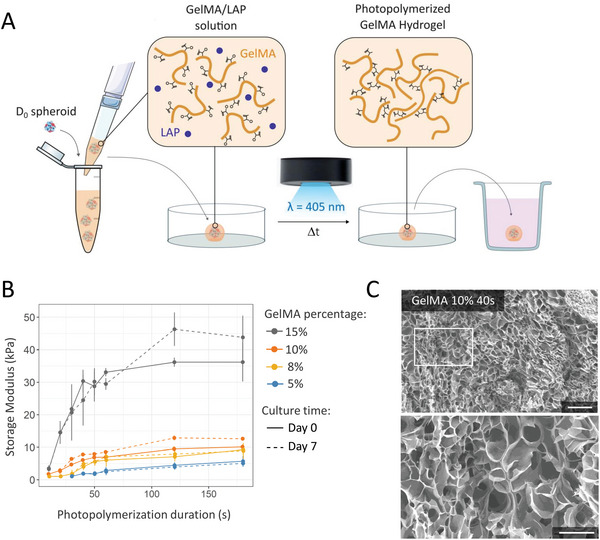
GelMA hydrogel processing and characterization of mechanical properties. A) Spheroid embedding in GelMA. Briefly, spheroids were collected and mixed with GelMA/0.1% LAP solution, then pipetted individually in a fixed volume of GelMA. Droplets containing spheroids were dispensed on an anti‐adhesive PDMS surface before crosslinking with 405 nm light. Photo‐crosslinked GelMA‐spheroid droplets were further collected for culture. B) Mechanical characterization of GelMA hydrogel depending on GelMA percentage (5%, 8%, 10%, and 15%) and photo‐polymerization duration by rheological testing. Storage moduli of the hydrogels were calculated according to their stress–strain curves (*n* = 5). Data were obtained once samples were formed (Day 0) and after samples were kept in PBS at 37 °C in a CO_2_ incubator for a week (Day 7). Data are shown as mean ± standard deviation. C) Scanning electron microscopy of GelMA 10% cross sections. Scales: 200 µm. Bottom image shows a higher magnification of the outlined area from the top image. Scales: 100 µm.

**Figure 4:**
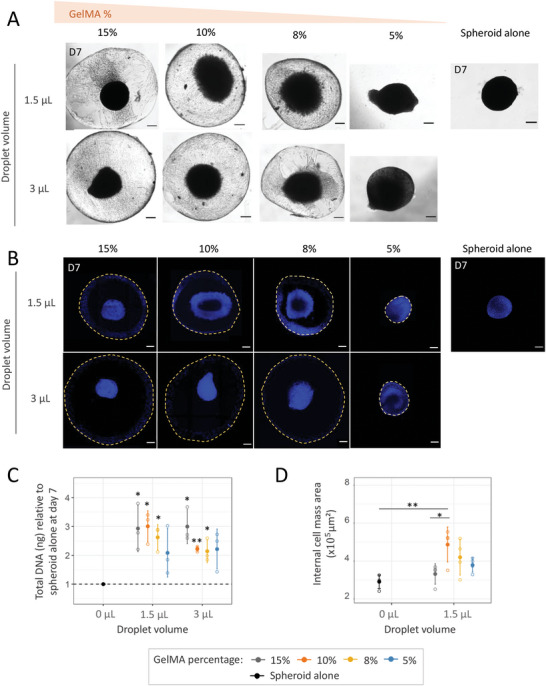
Optimal GelMA embedding parameters to promote cell mass expansion. P0‐SVF spheroids embedded in 3 or 1.5 µL of GelMA (15%, 10%, 8%, and 5%) were maintained for 7 days in EGM2 proliferation medium. Spheroids maintained without GelMA (spheroid alone) were used as controls. A) Representative brightfield images of whole GelMA‐spheroid droplets and spheroids alone at day 7. B) Immunofluorescence confocal images of DAPI staining (cell nuclei) to assess spheroid morphology depending on GelMA percentage and droplet volume. Images are z projections of confocal slices, from top to bottom, of spheroids inside the hydrogel. Yellow dashed lines highlight the delimitation of the GelMA droplet. C) Average DNA content per embedded spheroid. Data are expressed relative to spheroid alone. D) Quantification of spheroid area inside hydrogel from confocal z‐projections. All quantitative values are shown as mean ± standard deviations. Statistical analysis was performed by one or two‐sample *t*‐tests. Statistical significances: ^*^ *p* ≤ 0.05 and ^**^ *p* < 0.01.

To find a suitable biomechanical environment ensuring cell mass expansion after embedding D0 spheroid inside GelMA (Figure **3**A), different parameters of hydrogel embedding were studied (Figures 3 and 4). GelMA stiffness was tuned by varying gel percentage and photopolymerization time (Figure 3). The stiffness of the hydrogel alone remained below 8 kPa, within the range of beige adipose tissue stiffness,^[^ ^43^ ^]^ when a GelMA percentage of up to 10% (w/v) and a 1 min cross‐linking time was used. The resulting substrate appeared stable after 7 days in culture (Figure 3B).

To generate stable reticulated GelMA droplets for spheroid embedding while limiting exposition to free radical and subsequent phototoxicity,^[^ ^44^, ^45^ ^]^ a short crosslinking duration (40 s) was privileged. This duration generated hydrogels with Young's modulus ranging from 2 to 30 kPa (5%: 1.97 ± 0.4, 8%: 4.02 ± 1.07, 10%: 6.11 ± 0.15, and 15%: 30.36 ± 3.49) (Figure 3B). Scanning electron microscope images of GelMA hydrogel cross‐sections highlighted the presence of interconnected and macro‐porous structures in 10% GelMA hydrogel photopolymerized for 40 s (Figure 3C).

In combination with GelMA stiffness, the hydrogel volume used for spheroid embedding was investigated to promote cell survival and expansion (**Figure** **4**A). Brightfield images revealed important hydrogel remodeling after 7 days of culture with GelMA 5%, especially with 1.5 µL (Figure 4A). Total DNA content was measured to account for total cell expansion (Figure 4C). Except for GelMA 5%, the DNA content was significantly higher in the presence of the hydrogel compared to spheroid alone, regardless of hydrogel volume. However, DNA content tended to be higher or equivalent to 1.5 µL compared to 3 µL (Figure 4C). Hence, 1.5 µL was identified as the optimal droplet volume to promote high DNA content independently of hydrogel composition (Figure 4C). In addition, the evolution of the initial spheroid was studied by evaluating the internal cell mass expansion area with DAPI staining (Figure 4D). Under 1.5 µL condition, the highest internal mass expansion was observed for 10% GelMA.the highest internal mass expansion was observed for 10% GelMA. Overall, these results identified 1.5 µL droplet volume and 10% GelMA as optimal GelMA embedding conditions. These conditions were used for subsequent experiments.

### 3.3 Differentiation After Cell Mass Expansion Generates Vascularized Human Beige Adipose Organoid using P0‐SVF

**Figure 5:**
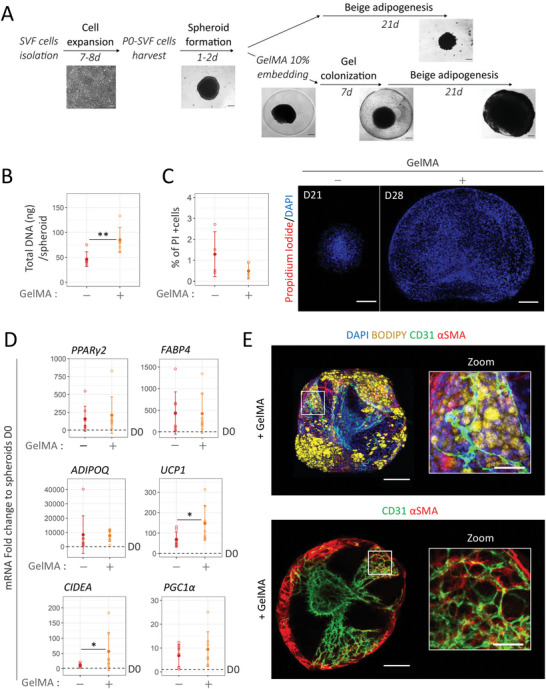
GelMA embedding promotes beige adipogenesis, vascular formation, and long‐term cell maintenance. A) Culture of adipose tissue organoids and spheroids derived from P0‐SVF cells. Spheroids embedded or not in GelMA 10% were analyzed after differentiation in an adipogenic medium containing SB431542. Scale bar: 200 µm. B) Average DNA content per spheroid (*n* = 6). C) Confocal imaging and quantification of propidium iodide (Dead cells in red) staining in regard to DAPI (blue) staining within spheroids. Scale bar: 200 µm. (‐GelMA: *n* = 4, +GelMA *n* = 3). E) Gene expression analysis of beige adipocytes markers (*UCP1*, *CIDEA*, and *PGC1α)* and generic adipocyte markers (*PPARγ2*, *FABP4*, and *ADIPOQ*) (*n* = 8). Quantification is expressed relative to non‐embedded undifferentiated spheroids (D0). F) Immunofluorescence analysis. Specific antibodies against human endothelial cell marker CD31 (green) and α‐smooth muscle actin (SMA) (red) were used. Lipids containing cells were revealed by bodipy staining. DAPI staining highlights cell nuclei. Scale bar: 200 µm. White squared images are zoomed areas. Scale bar: 50 µm. All statistical analyses were performed by a two‐sample *t*‐test. Statistical significance: ^*^ *p*≤ 0.05 and ^**^ *p* < 0.01.

The effect of GelMA embedding on long‐term maintenance and differentiation of P0‐SVF cells was investigated (**Figure** **5**A). GelMA embedding promoted the generation of tissue constructs with increased cell number compared to non‐embedded spheroids, as demonstrated by DNA quantification (Figure 5B). Under these conditions, almost no dead cells were detected by PI staining (Figure 5C). Interestingly, this result was also true in the absence of TGFβ inhibition (Figure S2A, Supporting Information).

Regarding beige adipocyte differentiation, P0‐SVF cells embedded in GelMA exhibited an increased expression of adipocyte markers such as *PPARγ2*, *FABP4*, and *ADIPOQ* during differentiation (D21 versus D0), comparable to spheroids without hydrogel embedding after differentiation (Figure 5D). Therefore, P0‐SVF spheroids embedded in GelMA maintained their adipocyte differentiation potential. It is noteworthy that the total amount of RNA per construct was higher in embedded versus non‐embedded spheroid (‐GelMA: 259.6 ± 105.0 ng per spheroid vs + GelMA: 101.4 ± 49.5 ng per spheroid, two‐sample *t*‐test: *p* = 0.002, data not shown) meaning that the total yield of adipocyte differentiation is the highest in the embedded spheroids. Interestingly, the expression of beige adipocyte markers, including *UCP1* and *CIDEA* was significantly higher in embedded conditions compared to spheroids cultured alone (Figure 5D). Immunofluorescence experiments after differentiation confirmed the presence of adipocytes stained by BODIPY (Figure 5E). In the absence of TGFβ inhibition, such adipogenesis induction was not observed with no BODIPY signal detected (Figure S2B, Supporting Information). Hence, GelMA embedding in combination with TGFβ inhibition promoted ASC differentiation toward the beige adipocyte phenotype while also increasing cell mass expansion. Within embedded spheroids, adipocytes were found in close proximity to the endothelial network revealed by endothelial CD31+ cells aligned with αSMA+ cells (Figure 5E). This arrangement contrasted with spheroids cultured alone where adipocytes and endothelial cell compartments were present at different locations within the spheroid, i.e., at the periphery and in the center, respectively (Figure 2E). Moreover, immunohistochemistry analyses highlighted ongoing vascular lumen formation within embedded spheroids delimited by CD31+ and CD146 + cells (Figure S6A, Supporting Information). These tubular structures were closely associated with αSMA+ cells (Figure S6B, Supporting Information) suggesting initiation of structural stabilization. Therefore, GelMA embedding promoted the generation of vascularized beige adipose organoids that more closely recapitulated in vivo‐like adipose tissue cell organization.

### 3.4 Human Beige Adipose Organoids Exhibit Metabolic and Secretory Functionality Following Activation by Canonical Beige Inducers

**Figure 6:**
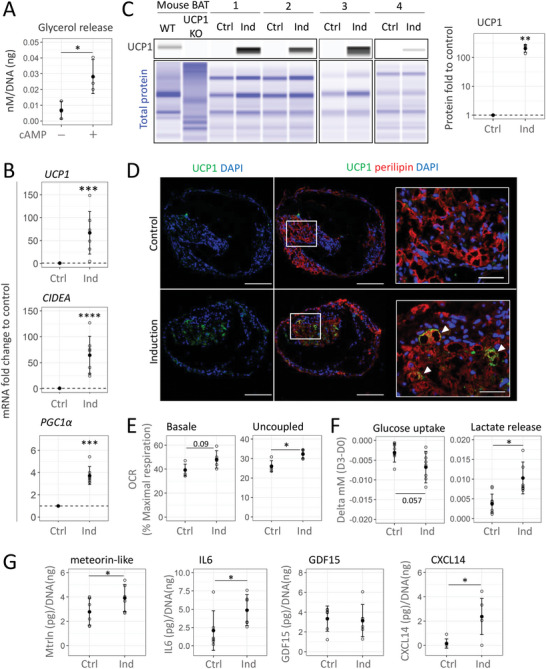
Activation of beige adipose organoids by canonical UCP1 inducers. Beige adipose organoids derived from P0‐SVF cells were treated (Ind) or not (Ctrl) with UCP1 inducers for the last three days of culture. A) Evaluation of lipolysis measured as glycerol release with or without stimulation (*n* = 3). B) Gene expression analysis of beige adipocytes markers (*UCP1*, *CIDEA*, and *PGC1α*). Fold changes are expressed relative to the Ctrl condition (*n* = 7). C) Expression of UCP1 protein under control (Ctrl) or induction (Ind) conditions derived from four different donors. Murine brown adipose tissue (BAT) from wild‐type (WT) and UCP1 KO mouse were used as positive and negative controls, respectively. Quantification of UCP1 expression was normalized by total protein signal and expressed as fold change to control condition (*n* = 4). D) Immunofluorescence images of UCP1 staining (green). Lipid‐containing cells were revealed by perilipin staining (red) and cell nuclei by DAPI staining. Scale bar: 200 µm. White squares show areas at higher magnifications where UCP1+ cells (white arrowheads) could be observed. Scale: 50 µm. E) Evaluation of basal (left) and uncoupled cell respiration (right) under control or induction conditions with Seahorse XF24 (*n* = 5). F) Measure of glucose uptake and lactate release during the three days of treatment (*n* = 6). G) Evaluation of meteorin‐like, IL‐6, GDF15, and CXCL14 secretions (*n* = 6). Measurements of glycerol and lactate release, glucose uptake, meteorin‐like, IL6, GDF15, and CXCL14 levels were normalized relative to DNA quantity/spheroid. All quantitative data are expressed as mean ± standard deviation. Statistical analysis of fold change to control was performed by one‐sample *t*‐test while statistical analysis for means comparisons was performed by paired two‐sample *t*‐test. Statistical significances: ^*^ *p* ≤ 0.05, ^**^ *p* < 0.01, ^***^ *p* < 0.01, and ^****^ *p* < 0.0001.

Next, the functionality of the vascularized beige adipose organoids resulting from these culture conditions was assessed. Treating differentiated embedded spheroids with cAMP significantly increased glycerol release within the supernatant, demonstrating that embedded spheroids show cAMP‐induced lipolysis (**Figure** **6**A). Treatment of differentiated embedded spheroids with a cocktail of canonical thermogenic inducers containing a cell‐permeable cAMP analog, i.e., 8‐CPT‐cAMP,^[^ ^46^ ^]^ rosiglitazone (PPARγ agonist),^[^ ^47^ ^]^ retinoid acid^[^ ^48^ ^]^ and thyroid hormone (T3),^[^ ^49^ ^]^ strongly increased *UCP1, CIDEA*, and *PGC1α* mRNA levels (Figure 6B) demonstrating the responsiveness of adipocytes in embedded spheroids to thermogenic pathway modulation. Embedded organoids were responsive to each inducer individually, but the combination of these inducers was the most efficient to promote significant upregulation of beige adipocyte markers (Figure S3, Supporting Information). This increase was concomitant with an increase in UCP1 protein content compared to untreated embedded spheroids where almost no UCP1 protein could be detected (Figure 6C; Figure S4, Supporting Information). The presence of UCP1 protein in perilipin‐expressing adipocytes was confirmed by immunofluorescence analysis (Figure 6D).

To further characterize metabolic function under activation conditions, the oxygen consumption rate (OCR) of differentiated organoids was quantified using seahorse technology. The contribution of basal respiration to maximal respiration tended to increase after UCP1 induction, suggesting that cells were metabolically closer to their maximal oxidative capacities (Figure 6E). Although additional cAMP did not further increase basal OCR, the contribution of uncoupled respiration to maximal respiration was significantly increased in response to the UCP1 cocktail of inducers (Figure 6E), as expected for mature beige adipocytes. Additionally, differentiated embedded spheroids exhibited a significant increase in lactate release after activation with the UCP1 cocktail inducers. In this condition, glucose uptake also tended to be increased but did not reach statistical significance (Figure 6F). These results are consistent with increased metabolic activity as expected from UCP1 induction.

Finally, the secretion of batokines, known to be released by activated brown/beige adipocytes^[^ ^3^ ^]^ such as meteorin‐like,^[^ ^50^ ^]^ IL6,^[^ ^51^, ^52^ ^]^ GDF15^[^ ^53^ ^]^ or CXCL14,^[^ ^54^ ^]^ were quantified within organoid supernatants. Levels of meteorin‐like, IL6, and CXCL14 were significantly increased in the supernatant of beige organoids activated with the cocktail of inducers compared to controls (Figure 6G), while GDF15 levels remained within the same range for both conditions. Altogether, these data clearly demonstrated that differentiated embedded spheroids displayed many metabolic and paracrine features of functional beige adipose tissue.

### 3.5 TGFβ Inhibition and GelMA Embedding Unlock Beige Adipogenesis in Vascularized Organoid from Native Human Stromal‐Vascular Fraction Cells

**Figure 7:**
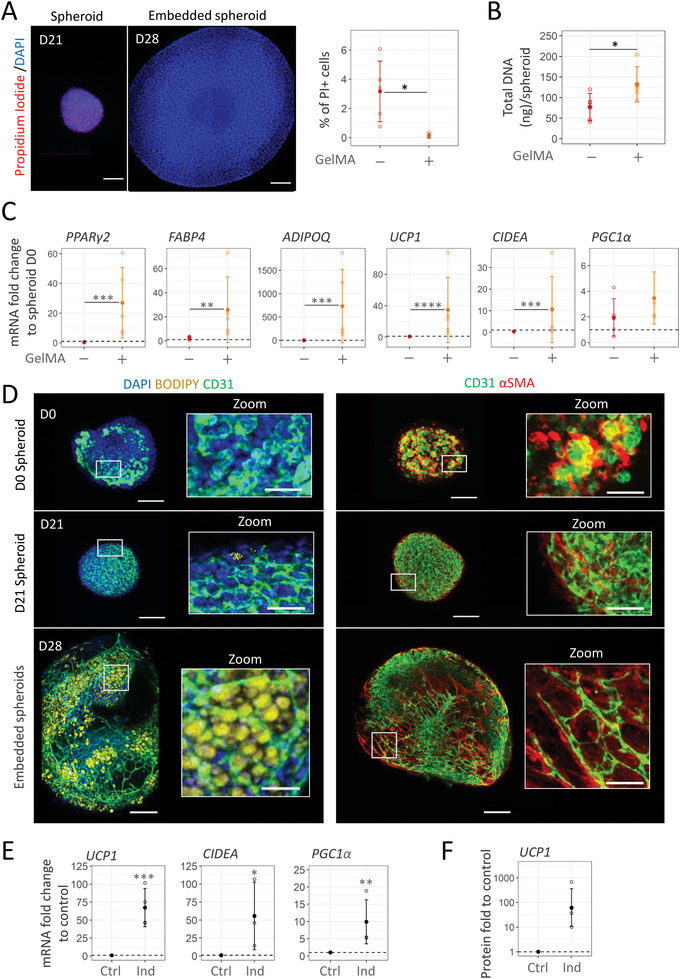
Combination of TGFβ inhibition and GelMA embedding unlocks beige adipogenesis and promotes vascular formation from SVF cells. A–E) Spheroids obtained directly from freshly isolated human stromal vascular fraction were embedded or not in GelMA 10% and analyzed at day 21 of differentiation in adipogenic medium with SB431542. A) Confocal images and quantification of propidium iodide (dead cells, red) staining relative to DAPI (blue) staining (*n* = 5). Scale bar: 200 µm. Statistical analysis was performed by two‐sample *t*‐tests. B) Average DNA content per spheroid with or without GelMA (*n* = 5). C) Immunofluorescence analysis. Specific antibodies against human endothelial cell marker CD31 (green) and α‐smooth muscle actin (SMA) (red) were used. Lipids containing cells were revealed by bodipy staining. DAPI staining highlights cell nuclei. White squares are zoomed areas showing endothelial cell organization. Scales: 200 µm. D) Gene expression analysis of beige adipocytes markers (*UCP1*, *CIDEA*, and *PGC1α)* and generic adipocyte markers (*PPARγ2*, *FABP4*, and *ADIPOQ*) in spheroids embedded or not in GelMA (*n* = 8) after 21 days of differentiation. Data are expressed relative to non‐embedded undifferentiated spheroids (D0). E‐G) To assess beiging potential, GelMA 10% embedded SVF spheroid were treated (Ind) or not (Ctrl) with UCP1 inducers for the last three days of differentiation. E) Gene expression analysis of brown adipocytes markers (*UCP1*, *CIDEA*, and *PGC1α*). Fold changes are expressed relative to controls (*n* = 3). F) Quantification of normalized UCP1 protein expression expressed as fold change relative to control condition (*n* = 3). All quantitative data are expressed as mean ± standard deviation. Statistical analysis of fold change to control was performed by one‐sample *t*‐test while statistical analysis for means comparisons was performed by paired two‐sample *t*‐test. Statistical significance: ^*^ *p* ≤ 0.05, ^**^ *p* < 0.01, ^***^ *p* < 0.01, and ^****^ *p* < 0.0001.

The SVF obtained directly after adipose tissue digestion contains heterogeneous cell populations comprising ASC, endothelial progenitors but also hematopoietic cells.^[^ ^55^ ^]^ SVF models, therefore, more closely recapitulate patient tissue heterogeneity than amplified P0‐SVF cells. The method for generating beige adipose tissue organoids was evaluated using SVF cells directly isolated from native adipose tissue. As observed with P0‐SVF cells, GelMA embedding significantly decreased SVF cell mortality after 21 days of differentiation compared to spheroids cultivated without hydrogel (**Figure** **7**A). Indeed, in the absence of GelMA, the percentage of dead SVF cells was even higher than in P0‐SVF spheroids at day 21 (SVF: 3.76% ± 1.82%, Figure 7A vs P0‐SVF: 1.29% ± 1.07%, Figure 5A, two‐sample *t*‐test: *p*  =  0.058). GelMA embedding also promoted more homogeneous and slightly increased DNA levels in embedded SVF spheroids compared to SVF spheroids cultured alone (Figure 7B).

In contrast to P0‐SVF cells, adipogenic medium containing the TGFβ pathway inhibitor was not sufficient to induce robust adipocyte differentiation from SVF cells in the three‐dimensional (3D) spheroid model without hydrogel, as revealed by low mRNA levels of adipocytes markers *FABP4*, *PPARγ2*, and *ADIPOQ* (Figure 7C) and by lack of BODIPY staining (Figure 7D).

Interestingly, combining GelMA embedding with TGFβ inhibition led to a significant increase in expression of adipogenesis and beige adipocytes markers (Figure 7C). Immunofluorescence analysis performed on differentiated embedded SVF spheroids revealed the presence of adipocytes in between CD31+ and αSMA+ cell networks (Figure 7D).

Next, the beiging potential of SVF beige adipose organoid was then assessed. Treatment of SVF organoids with the UCP1 induction cocktail led to a significant increase in the expression of beige adipocyte markers *UCP1*, *CIDEA*, and *PGC1α* (Figure 7E). An increase of UCP1 at the protein level was also detected in SVF organoids with induction compared to controls (Figure 7F), although its overall expression remained lower than with P0‐SVF cells (Figure S5, Supporting Information). These results validated the feasibility of generating adipose organoids containing UCP1‐expressing cells directly from patient tissue cells (SVF cells) using this newly developed GelMA embedding engineering approach (Figure 1).

### 3.6 From Beige Adipose Organoid Generation to Micro‐Tissue Generation

**Figure 8:**
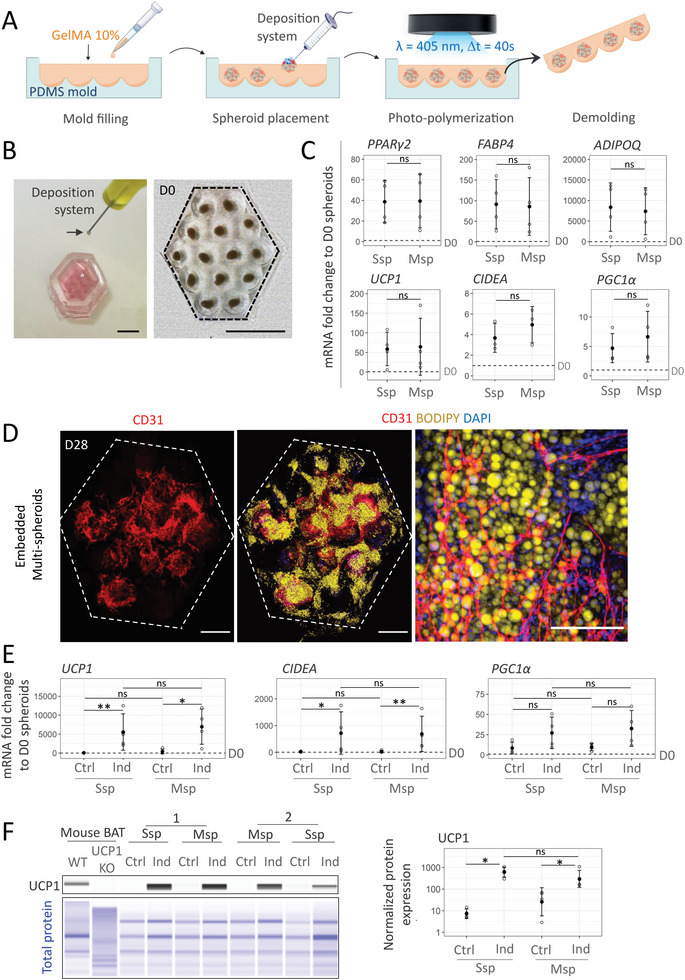
Generation of vascularized beige adipose micro‐tissue by multi‐spheroid assembly in GelMA. A) Engineering of multi‐spheroid tissue constructs. B) Macroscopic view of spheroid deposition in the PDMS mold and GelMA multi‐spheroid construct after demolding. Dotted lines highlight the contours of the GelMA construct. Scale: 2.5 mm. C) Gene expression analysis of beige adipocytes markers (*UCP1*, *CIDEA*, and *PGC1α)* and generic adipocyte markers (*PPARγ2*, *FABP4*, and *ADIPOQ*) in individually embedded spheroids (1sp) compared to multi‐spheroid construct (14 sp) generated from P0‐SVF cells, at the end of the culture process. Fold change relative to non‐embedded undifferentiated spheroids (D0) is shown (*n* = 4). Statistical analysis was performed by a two‐sample *t*‐test. D) Immunofluorescence images of lipids‐containing cells and endothelial cells revealed by BODIPY (yellow) and CD31 (red) stainings, respectively, within embedded multi‐spheroid P0‐SVF construct at the end of the culture process. DAPI staining highlights cell nuclei. Scale bar: 1000 µm. The right image depicts a magnified area. Scale bar: 200 µm. E–G) Assessment of embedded multi‐spheroid construct (14sp) response to UCP1 inducers in comparison to individually embedded spheroids (1sp). For each type of construct, cells were treated or not (Ind versus Ctrl) with UCP1 inducers for the last three days of culture. E) Gene expression analysis of beige adipocytes markers (*UCP1*, *CIDEA*, and *PGC1α*). Fold changes are expressed relative to non‐embedded undifferentiated spheroids (D0) (*n* = 4). Statistical analysis was performed by one‐way ANOVA followed by Tukey's comparison. F) UCP1 protein expression of multi‐spheroid (14 sp) and individual embedded spheroid (1sp) under control and induction conditions derived from two different donors. Wild type (WT) and UCP1 KO mouse brown adipose tissue (BAT) were used as positive and negative controls, respectively. Quantification of UCP1 expression normalized to total protein signal (*n* = 4). Statistical analysis was performed by Kruskal Wallis analysis followed by paired wise Wilcoxson test with Bonferoni correction. All quantitative data are expressed as mean ± standard deviation. Statistical significances: ns. non‐significant, ^*^ *p* ≤ 0.05 and ^**^ *p* ≤ 0.01.

The beige organoid method was next translated to the macro‐scale level using P0‐SVF cells to engineer beige adipose micro‐tissue at the centimetric scale. For this purpose, we introduced a guided‐assembly approach of multiple D0 spheroids in GelMA using micro‐fabricated PDMS molds composed of 14 cavities (**Figure** **8**A). This mold was specifically designed to preserve the GelMA volume per spheroid established for beige adipose organoid generation in Section 2.2. Thanks to a syringe‐based aspiration system, D0 spheroids were successfully deposited in the center of the cavities before photo‐polymerization and complete removal of the construct from the mold (Figure 8B). In the same way as embedded single spheroids (Ssp), the resulting embedded multi‐spheroid constructs (Msp) were allowed to proliferate for 7 days followed by 21 days of differentiation in adipogenic medium containing SB431542. An increase in tissue construct size did not impact cell mortality compared to single embedded spheroids as revealed by propidium iodide staining (Ssp: 0.49% ± 0.38% Figure 5C vs Msp: 0.37% ± 0.14%, *n* = 3, Figure S7A, Supporting Information, two‐sample *t*‐test: *p* = 0.61).

The expression of both adipogenic and beige adipocyte markers in embedded multi‐spheroid constructs (Msp) derived from P0‐SVF cells was consistent with an equivalent number of embedded single spheroids (Ssp) (Figure 8C). In addition, BODIPY staining revealed the abundant presence of adipocytes on most of the tissue construct, surrounded by a continuous network of CD31+ cells connecting different cavities (Figure 8D). As observed in embedded single spheroids, these CD31+ cells were frequently aligned with αSMA+ cells (Figure S7B, Supporting Information). This organization recapitulates in vivo‐like adipose tissue structure. To further confirm the beige adipocyte identity of the cells, differentiated embedded multi‐spheroids were treated with UCP1 inducers. Again, consistent with embedded spheroids, the UCP1 induction cocktail reproducibly led to the increased gene expression of beige adipocyte markers in treated multi‐spheroids compared to control embedded multi‐spheroids (Figure 8E). Increased UCP1 expression was further confirmed at the protein level (Figure 8F) in a comparable manner to individually embedded spheroids. Taken together, these results demonstrate that GelMA embedding can be scaled from single to multiple spheroids allowing the generation of centimetric size tissue constructs without hindering beige adipocyte differentiation. Moreover, this approach resulted in the generation of a pre‐vascularized beige adipose micro‐tissue. Importantly, the initial shape and size (20 mm^2^) of the beige adipose micro‐tissue were mostly maintained, even after 28 days of culture, allowing easy handling for potential biomedical and research applications.

## 4 Discussion

Relevant models of human beige adipose tissue are critical to better understand beige adipocyte physiology, a question of increasing importance in light of metabolic disorders^[^ ^2^ ^]^ but also as described in aging.^[^ ^9^, ^10^ ^]^ Human 3D adipocyte cultures have been shown to recapitulate the phenotype of mature white adipocytes.^[^ ^15^, ^16^, ^17^, ^18^, ^20^ ^]^ However, the field still lacks a system that truly recapitulates human beige adipose tissue biology, particularly the plasticity of this cell type. The few existing 3D‐engineered human beige adipose tissue models focus on beige adipogenesis and either lack the vascular compartment,^[^ ^21^, ^22^ ^]^ known for its importance in beige adipose tissue physiology,^[^ ^56^ ^]^ or are based on vascular explant culture.^[^ ^23^ ^]^ Here, we developed a modular and scalable engineering process that generates functional mature vascularized human beige adipose tissues through guided differentiation and self‐organization of human adult primary cells. Researchers could tailor the system for applications ranging from basic research to clinical uses.

Across tissue scales, exposure to beige adipogenic medium promoted the development of GelMA‐embedded spheroids into pre‐vascularized human beige adipose organoids that display functional features specific to beige adipose tissue. These organoids responded to canonical beige inducers with the significant upregulation of specific markers such as UCP1 mRNA and protein. In addition, the embedded organoids showed higher uncoupled respiration rates associated with the improvement of glucose uptake and lipolytic activity, key parameters for thermogenic activity.^[^ ^57^, ^58^ ^]^ Moreover, upon activation, the organoids recapitulated increased secretion of paracrine factors such as meteorin‐like, CXCL14, or IL6, which are essential for the beneficial effects of brown/beige adipose tissues on systemic metabolism.^[^ ^12^ ^]^ In addition, organoids contained a self‐organized vascular system in close proximity to adipocytes, as found in vivo. This organization was associated with an improved commitment toward the beige adipocyte lineage compared to differentiated spheroids where such vascular proximity was not observed. These data are consistent with previous studies demonstrating a correlation between angiogenesis and increased plasticity of adipose tissue toward the beige phenotype, highlighting the importance of sustaining both the vascular and adipocyte compartments for a fully relevant beige adipose tissue in vitro model.^[^ ^59^, ^60^ ^]^ Mural cells, including perivascular smooth muscle cells and pericytes, are both important components of vascular tube formation through vessel stabilization^[^ ^61^, ^62^ ^]^ and potential mesenchymal stem cell niches.^[^ ^63^ ^]^ Importantly, pseudo‐vascular networks within organoids possess the ability to undergo vascular tubule formation that is aligned with αSMA expressing cells occupying a pericellular position akin to perivascular smooth muscle cells. These data suggest vessel structural maturation. However, these structures seem to be still under development as only a few cells expressing other canonical pericytes markers (CD146,^[^ ^64^ ^]^ Platelet derived growth factor receptor PDGFRβ,^[^ ^65^ ^]^ Figure S6B, Supporting Information) were found close to endothelial cell networks.

This complex phenotype in culture, which resembles native tissue organization, results from a combination of the optimized biomaterial we have used, the conditions of its use, and the culture media we have developed.

Generation of organoids from various organs often relies on the use of Matrigel®,^[^ ^25^, ^26^, ^28^ ^]^ a mouse tumor extracellular matrix extract that is ill‐defined and whose biomechanical features are not tunable and incompatible with good manufacturing practice (GMP)‐compliant procedure. GelMA with its tunable mechanical properties close to those of adipose tissue and its excellent biocompatibility^[^ ^31^ ^]^ appears to be a promising alternative for engineering soft collagen‐rich tissue such as adipose tissue.^[^ ^66^ ^]^ Furthermore, the very recent development of GMP‐grade GelMA definitively opened up its use for putative clinical applications (Rousselot® Biomedical).

The optimal combination of volume per spheroid, percentage, and reticulation of GelMA embedding conditions is important to support cell mass expansion contributing to the development of beige adipose organoids. Defining this combination was critical to translating our engineering process from single to multiple spheroid assemblies. We tuned GelMA concentrations (5–10%) to generate substrates of known biochemical composition and a Storage's modulus between 1.97 and 6.11 kPa that matches the stiffness range of white (≈1–3 kPa)^[^ ^67^ ^]^ and beige (≈4–6 kPa)^[^ ^43^ ^]^ adipose tissue. Maximum cell expansion and viability inside hydrogel were found for 10% GelMA and a droplet volume of 1.5 µL. A GelMA percentage below 10% showed a higher variability and collapse of the hydrogel on itself, especially for 5% GelMA substrates supporting tissues with a lower DNA content. This GelMA collapse would result from increased degradation kinetics and lower stiffness favoring contraction during cell proliferation.^[^ ^68^ ^]^

This study highlights the importance of defining a physicochemical environment with proper mechanical stability and porosity that allows cells to expand over time and self‐organize at various tissue scales.

Self‐organized vascular formation in close proximity to adipocytes was achieved via the refinement of our previously published medium.^[^ ^18^ ^]^ Inhibition of the TGFβ pathway has been shown to favor the differentiation of adipose progenitors toward adipogenesis,^[^ ^69^ ^]^ including toward the beige lineage.^[^ ^70^ ^]^ Furthermore, such inhibition decreases ASC differentiation into myofibroblasts. This observation is consistent with a study from Di Stefano et al. demonstrating that ASC spheroids display increased TGFβ expression compared to 2D culture which suggests higher activation of the pro‐myofibrolastic TGFβ pathway.^[^ ^71^ ^]^ As TGFβ also inhibits vascular formation,^[^ ^72^ ^]^ the use of SB431542, a TGFβ inhibitor, is a viable alternative in 3D culture to promote beige adipogenesis while maintaining endothelial cell network. Remarkably, we found a combined positive effect of TGFβ inhibition and GelMA embedding on beige adipocyte commitment of amplified SVF cells. This effect was even more potent with crude SVF cells where a defined combination of chemical and mechanical cues was a necessary and sufficient condition to unlock beige adipogenesis. The physiological distribution of endothelial cells in between adipocytes suggests an increased ability of cells into GelMA hydrogel to self‐organize compared to unembedded spheroids. The emergence of these pseudo‐vascular networks from the internal cell mass within organoids implied increased angiogenic potential of ASCs in close contact with cell aggregates as previously described.^[^ ^73^ ^]^ In addition, unstained cells from immunofluorescence analyses revealed non‐adipocytes and non‐vascular cell compartments which could suggest the presence of undifferentiated progenitor cells. Further extensive investigations will be required to better characterize organoids' cell composition. The presence of progenitor cells in the organoid such as found in the native tissue could be relevant for its long‐term cell maintenance.

Simultaneous embedding of pre‐assembled spheroids using a molding approach represents a simple, practical, and rapid way to generate microtissue with variable sizes. Tuning of a single factor, i.e., matrix volume to spheroid ratio, is sufficient to achieve comparable phenotypes in tissues grown at a micro and macro scale. Besides, the beneficial effect of GelMA embedding on cell spheroid viability is maintained with increasing tissue size. This is especially important as the main challenge of large‐scale bio‐fabrication is to maintain an adequate supply of nutrients/oxygen and avoid cell viability issues. These data suggest that it may be possible to construct custom tissues that meet specific cell requirements by using the matrix‐to‐cell ratio as a scaling factor. Generating centimeter‐sized tissue efficiently is particularly crucial when aiming to translate studies on beige adipose tissue transplantation from mice^[^ ^11^, ^12^, ^13^, ^14^ ^]^ to biomedical applications in obese or diabetic patients. In this context, the successful implantation of a substantial number of cells is necessary to potentially achieve therapeutic effectiveness. Additionally, these beige adipose micro‐tissues remained easy to handle at the end of the culture process facilitating surgical implantation and displayed embedded vasculature that may help to sustain micro‐tissue integration and viability after implantation. Moreover, it is reasonable to assume that a micro‐tissue of significant size would be less sensitive to the effect of the environment, and would retain the desired function for a longer period after transplantation.

Regardless of tissue size, cell sources should also be a modulable parameter to consider in organoid applications. As discussed above, the promise of cell‐based therapies comes with several challenges including obtaining sufficient cells to display significant therapeutic efficacy with consistent cell quality. Ex vivo expansion of SVF cells, such as P0‐SVF cells, allows the production of a large number of relatively homogeneous ASCs compared to native SVF. P0‐SVF cells produced organoids with the highest thermogenic potential regarding total UCP1 protein quantity and metabolic functionality, making them a preferred cell source for therapeutic implantation or high throughput drug screening. However, changes in expression profile and functional drift can be observed from ASCs amplification.^[^ ^74^, ^75^ ^]^ Therefore, despite their lower thermogenic potential, organoids generated from crude SVF represent a promising tool for precision medicine with personalized drug screening as they are more susceptible to conserve native cell heterogeneity and hallmarks of patient metabolism as found in vivo. In conclusion, we propose a convenient process for the generation of functional vascularized 3D human beige adipose organoids and micro‐tissue through the definition of a permissive microenvironment and its translation to a macroscale level. We demonstrated that fully functional vascularized beige adipose organoids containing inducible adipocytes could be generated from both amplified and native SVF cells. Such models will help to elucidate the development and function of a tissue that is difficult to access in adult humans while reducing animal use. Indeed, organoids could be used to better understand the underlying mechanisms of beige tissue activation in physiological or pathophysiological conditions. Generating patient‐derived organoids can be easily considered for disease modeling and drug screening. Therefore, we believe that these models will help to efficiently select relevant targets and compounds for therapeutic use in humans, thus bridging the gap between the bench and the bedside. Through their scalability, these new human beige adipose tissue models open the door to more relevant in vitro studies for basic research and a wide range of therapeutic applications from bioassays to biotherapies.

## Conflict of Interest

L.C. Is co‐founder, shareholder and discloses consultancy work for Cell‐Easy company. L.C., L.V., L.M., G.E., C.D., A.C., M.E. have filed 2 patents (EP22306953, EP 233051366) related to this work. All other co‐authors display no conflict of interest.

## Author Contributions

M.E. and L.V. contributed equally to this work and are co‐first authors. G.E., L.V., L.B., C.V., and L.M. designed the GelMA construct. M.E. and L.V. conducted cell experiments and performed functional assays. M.P. and F.V. performed ELISA experiments to determine paracrine activity. Y.J. contributed to metabolic studies. F.D. designed the initial media culture. I.R.L. and M.P. performed immunochemistry experiments to identify perivascular cells. S.B. and X.Y. participated to cell culture. B.C. provided human cells. M.E., L.V., I.R.L., A.C., L.M., and L.C. analyzed the data and wrote the manuscript. L.V., C.D., A.C., L.M., and L.C. designed and supervised the research. L.V., C.D., A.C., L.M., and L.C. designed the research. AC, LM and LC contributed equally to the supervision of this work and are co‐last authors. All authors revised and approved the final version of the manuscript.

## Supporting information

Supporting Information

## Data Availability

The data that support the findings of this study are available from the corresponding author upon reasonable request.
