# Supplementary material for: Scalable Generation of Pre‐Vascularized and Functional Human Beige Adipose Organoids
Source: Adv Sci (Weinh). 2023 Sep 20;10(31):2301499. doi: 10.1002/advs.202301499 (PMC10625054; doi:10.1002/advs.202301499)
Supplement: Supplementary file 1 — Supporting Information [file ADVS-10-2301499-s001.pdf]

ADVANCED SCIENCE
Open Access

Supporting Information

for *Adv. Sci.*, DOI 10.1002/advs.202301499

Scalable Generation of Pre-Vascularized and Functional Human Beige Adipose Organoids

*Mélanie Escudero, Laurence Vaysse, Gozde Eke, Marion Peyrou, Francesc Villarroya, Sophie Bonnel, Yannick Jeanson, Louisa Boyer, Christophe Vieu, Benoit Chaput, Xi Yao, Frédéric Deschaseaux, Mélissa Parny, Isabelle Raymond-Letron, Christian Dani, Audrey Carrière, Laurent Malaquin and Louis Casteilla**

Supporting Information

Scalable Generation of Pre-Vascularized and Functional Human Beige Adipose Organoids

Mélanie Escudero§[1, 2] Laurence Vaysse§[1], Gozde Eke[2], Marion Peyrou[3], Francesc Villarroya[3] Sophie Bonnel[1], Yannick Jeanson[1], Louisa Boyer[2], Christophe Vieu[2], Benoit Chaput[4], Xi Yao[5], Frédéric Deschazeaux[1], Mélissa Parny[1,6], Isabelle Raymond-Letron[1,6], Christian Dani[5], Audrey Carrière[1], Laurent Malaquin*[2], Louis Casteilla[1]**

Immunohistochemistry protocol:

Embedded spheroids were fixed overnight in 4% buffered formalin for 24h before storage in PBS at 4°C. After paraffin embedding, 3µm thickness paraffin sections were dewaxed (successive toluene and descending alcohols bathes) and stained with haematoxilin and eosin. CD31 and CD146 labelling of 3-µm serial sections of paraffin-embedded blocks were performed out after antigen retrieval (Ptlink high pH, reference K800021-2, Dako for CD31 labelling and Ptlink low pH, reference K8005, Dako for CD146 labelling) for 30 min at 96°C. Anti-human CD31 murine monoclonal antibody (M0823, clone JC70A, dilution 1:120) or CD146 rabbit monoclonal antibody (ab75769, Abcam, dilution 1:250) were incubated for 50 min at room temperature. Staining was carried out with EnVision Flex (reference K8000, Dako) for 30 min at room temperature, followed by DAB as a chromogen (reference K8000, 10 min room temperature). Slides were then counterstained with hematoxylin (reference C0283, DIAPATH). The stained slides were scanned (Panoramic Desk, 3D Histec) or imaged by light microscopy on a Nikon Eclipse Ci-L microscope with a DS138 Fi3 Camera and NIS Elements D software.

Table S1: List of primer used for qPCR and qRT-PCR analyses.

Gene		Sequence (5'-3')
RPLP0	Forward	CGTCCTCGTGGAAGTGACAT
	Reverse	TAGTTGGACTTCCAGGTCGC
GUSB	Forward	AGCCAGTTCCTCATCAATGG
	Reverse	GGTAGTGGCTGGTACGGAAA
PPIA	Forward	GCCGAGGAAAACCGTGTACTAT
	Reverse	TCTTTGGGACCTTGTCTGCAA
YWAZ	Forward	AGCAGGCTGAGCGATATGAT
	Reverse	TCTCAGCACCTTCCGTCTTT
FABP4	Forward	AAACTGGTGGTGGAATGCGT
	Reverse	GCGAACTTCAGTCCAGGTCA
PPARGγ2	Forward	GATACACTGTCTGCAAACATATCA
	Reverse	CACGGAGCTGATCCCAA
ADIPOQ	Forward	CAGAGATGGCACCCCTGGTG
	Reverse	TTCACCGATGTCTCCCTTAG
UCP1	Forward	GTGTGCCCAACTGTGCAATG
	Reverse	CCAGGATCCAAGTCGCAAGA
CIDEA	Forward	AGTCCTGTTGACCCCGCTC
	Reverse	GCTATTCCCGACCTCTTCGG
PGC1-α	Forward	CCGCACGCACCGAAA
	Reverse	TCGTGCTGATATTCCTCGTAGCT
CD31	Forward	GGAAAGCTGTCCCTGATGC
	Reverse	CATCTGGCCTTGCTGTCTAA
vWF	Forward	GATGGAGTCCAGCACCAGTT
	Reverse	GCTACTTCACACAGGCCACA
αSMA	Forward	CTATGCCTCTGGACGCACAACT
	Reverse	CAGATCCAGACGCATGATGGCA
COL1	Forward	GCTAACCCCCTCCCCAGCCA
	Reverse	GAGCAGGAGCCGGAGGTCCA
CTGF	Forward	TGCCCGGGAAATGCTGCGAG
	Reverse	CAGTCGGTAAGCCGCGAGGG

Table S2. List of antibodies and dyes used for immunofluorescence cell characterization.

Type	Name	Dilution	Company	Cat. No.
Primary antibodies	Anti-Human CD31 Rabbit monoclonal (Clone: EP78)	1:200	Epitomics (Burlingame, USA)	AC-0083A
	Anti-Human CD31 Mouse monoclonal (Clone: JC70A)	1:200	Dako-Agilent (Paris, France)	M0823
	Anti-perilipin Guinea pig polyclonal	1:100	Progen (Heidelberg,Germany)	GP29
	Anti-αSMA mouse monoclonal (Clone: 1A4)	1:100	Dako-Agilent (Paris, France)	M0851
	Anti-human/mouse UCP1 monoclonal mouse IgG_{2B}	1:500	R&D Systems Biotechne (Mineapolis, USA)	MAB6158
	Anti-CD146 Rabbit monoclonal	1:200	Abcam (Cambridge, UK)	ab75769
	Anti-PDGFRβ Rabbit polyclonal	1:50	DiagOmics (Blagnac, France)	A2180-20
Secondary antibodies	Alexa Fluor 488 Goat anti-rabbit IgG	1:500	Life technologies (Paisley, UK)	A11008
	Alexa Fluor 594 Goat anti-rabbit IgG	1:500	Life technologies (Paisley, UK)	A21207
	Alexa Fluor 647 Goat anti-Guinea Pig IgG Highly Cross-Adsorbed	1:500	Life technologies (Paisley, UK)	A21450
	Alexa Fluor 594 Goat anti-mouse IgG	1:500	Life technologies (Paisley, UK)	A11005
Dyes	BODIPY™ 493/503 (4,4-Difluoro-1,3,5,7,8-Pentamethyl-4-Bora-3a,4a-Diaza-*s*-Indacene)	1:500	Life technologies (Paisley, UK)	D3922
	DAPI (4',6-Diamidino-2-Phenylindole, Dihydrochloride)	1:5000	Life technologies (Paisley, UK)	D1306
	Propidium Iodide solution in water 1.0 mg/mL	1:100	Life technologies (Paisley, UK)	P3566

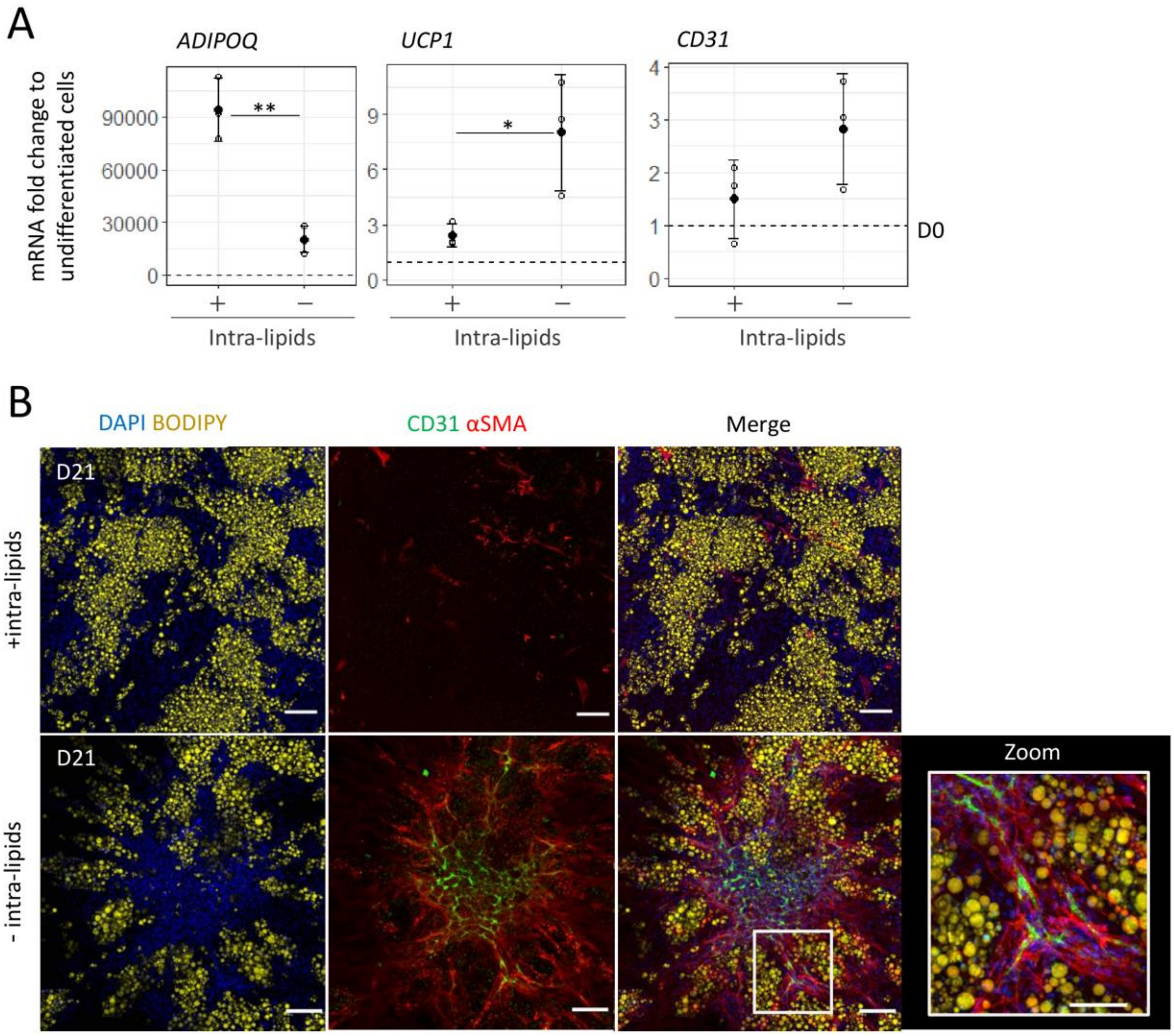

Figure S1. Development of a beige adipogenic cocktail that preserves endothelial cells from P0-SVF cells. P0-SVF cells were maintained in 2D culture for 21 days of differentiation either in an adipogenic cocktail containing intralipids or deprived of intralipids. A) Gene expression analysis of *UCP1* beige adipocytes marker, *ADIPOQ* generic adipocyte marker, and *CD31* endothelial cell marker. Fold changes are expressed relative to undifferentiated cells (D0). Data are expressed as mean ± standard deviation of three independent experiments from different human donors. Statistical analysis was performed by two sample t-test. *$p<0.05$, ** $p<0.01$. B) Immunofluorescence analysis. Specific antibodies against human endothelial cell marker CD31 (green) and α-smooth muscle actin (SMA) (red) were used. Lipids containing cells were revealed by bodipy staining. DAPI staining highlights cell nuclei. White square is a zoomed area showing endothelial cells organization aligned with αSMA+ cells in between adipocytes clusters derived from P0-SVF cells under deprived intralipid conditions (Scale bar: 100 µm).

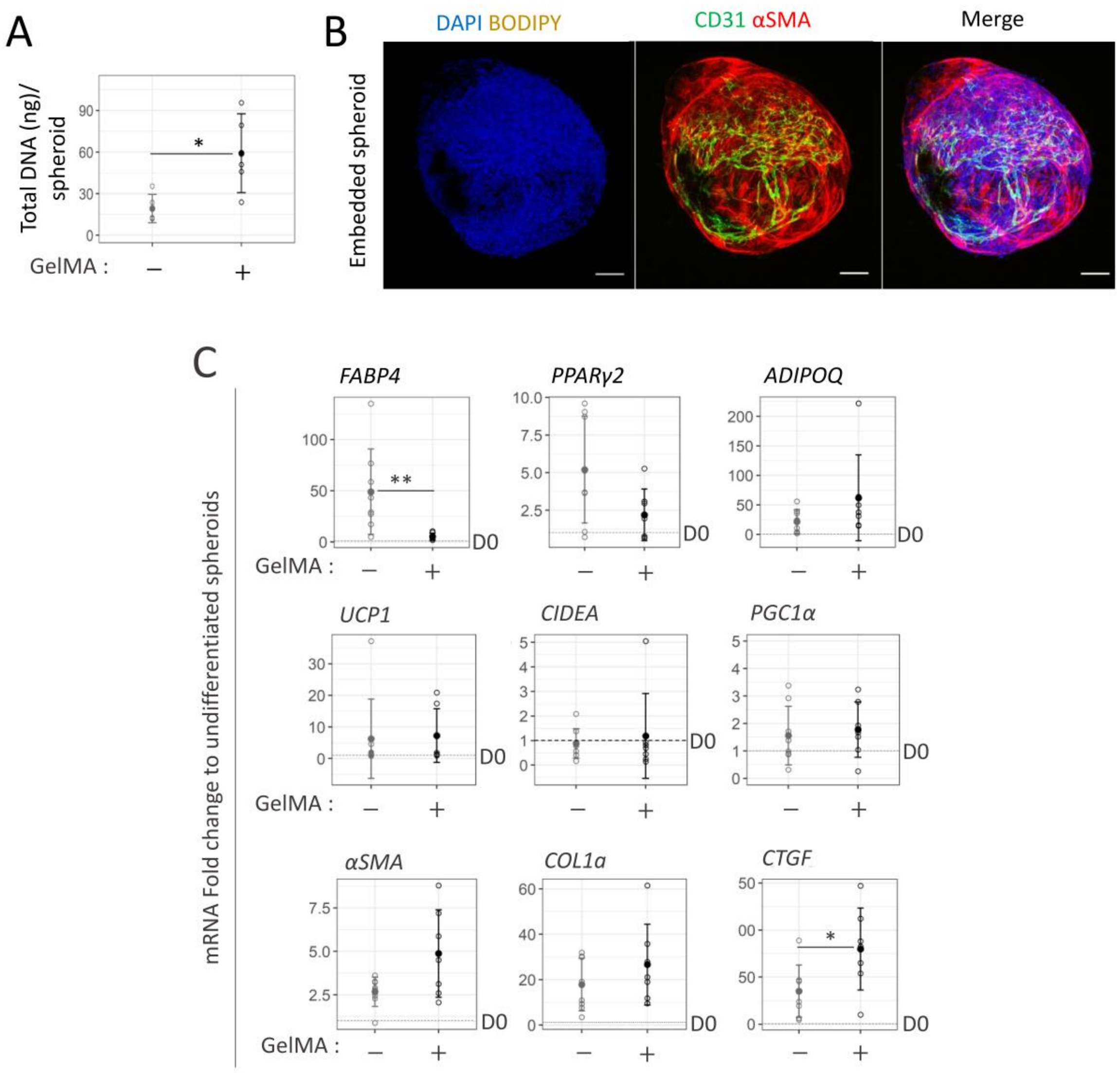

Figure S2. GelMA 10% embedding promotes cell expansion and vascular formation from P0-SVF cells but is not sufficient to induce adipogenesis in absence of TGFβ inhibition. P0-SVF spheroids embedded or not in 10% GelMA were analyzed after differentiation in adipogenic medium. A) Average DNA content per spheroid (*n* = 6). B) Immunofluorescence images of lipid-containing cells, endothelial cells and pericytes revealed by BODIPY (yellow), CD31 (green) and αSMA (red) stainings respectively. DAPI staining highlights cell nuclei. Scale bar: 200 µm.C) Gene expression analysis of beige adipocyte markers (*UCP1*, *CIDEA*, *PGC1a)*, generic adipocyte markers (*PPARγ2*, *FABP4*, *ADIPOQ*) and myofibroblast markers (*αSMA*, *COL1a*, *CTGF*).

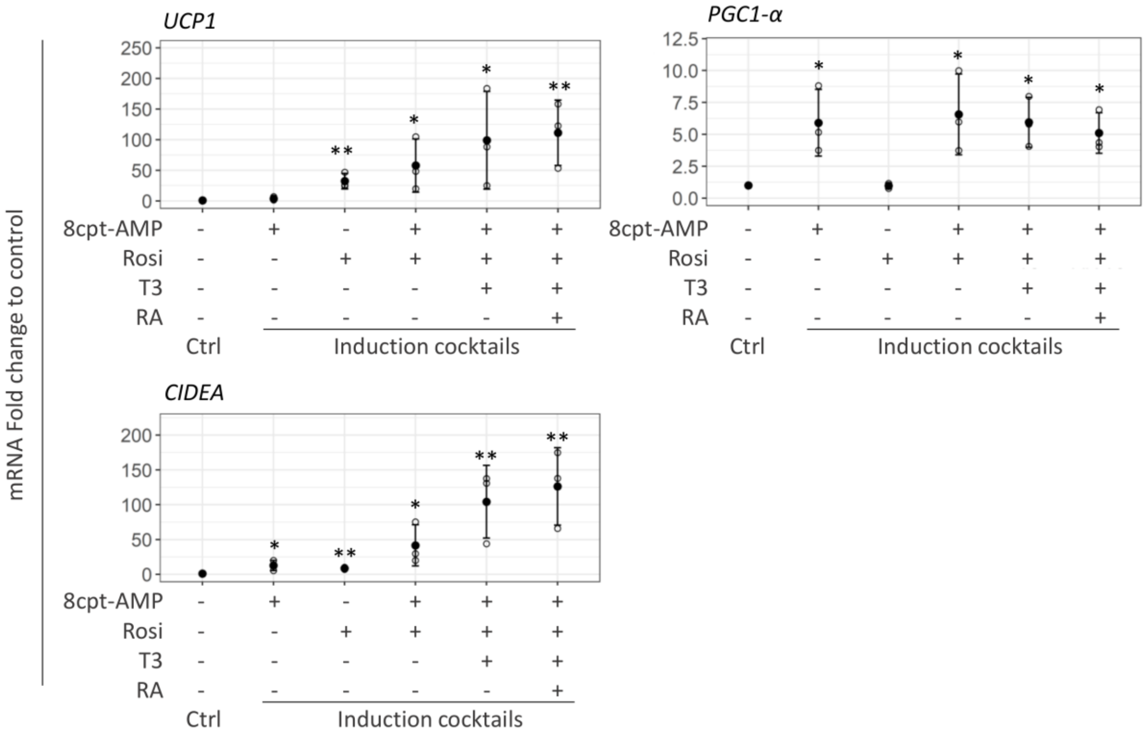

Figure S3. **Gene expression response of P0-SVF beige adipose organoids to combination of UCP1 inducers.** Beige adipose organoids derived from P0-SVF cells were treated (induction cocktails) or not (Ctrl) with combinations of UCP1 inducers for the last three days of culture. 8cpt-AMP: 200 µM, rosiglitazone (Rosi): 1 µM, triiodothyronine (T3): 0.2 nM, all-trans retinoid acid (RA): 0.1 µM. Analysis of brown adipocytes markers (*UCP1*, *CIDEA*, *PGC1α*). Fold changes are expressed relative to controls (n=3). All quantitative data are expressed as mean +/- standard deviation. Statistical analysis of fold change to control was performed by one sample t-test. Statistical significance: * $p \leq 0.05$, ** $p < 0.01$

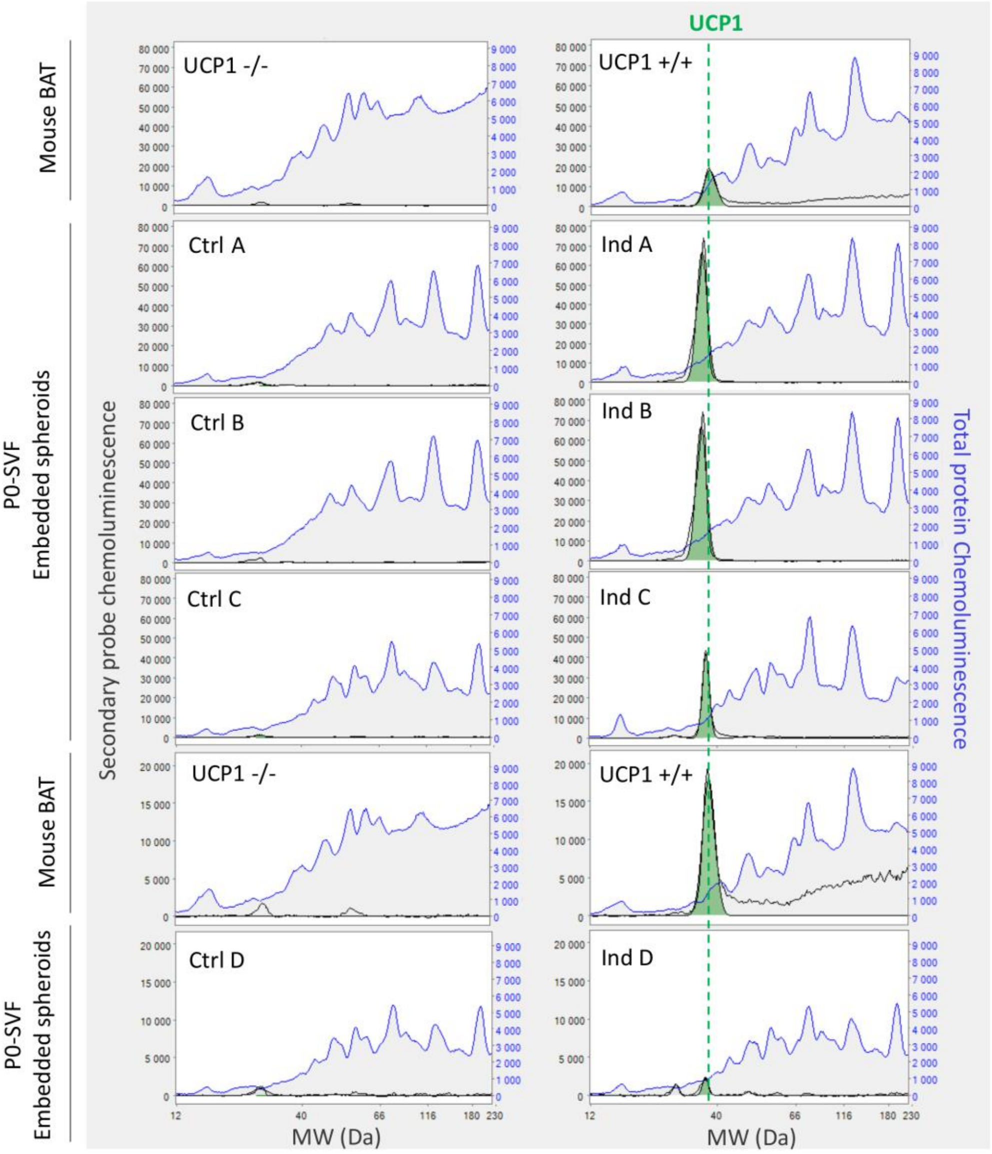

Figure S4. Characterization of UCP1 expression in P0-SVF organoids by proteinSimple capillary electrophoresis immunoassay under control (Ctrl) or induction (Ind) conditions. Brown adipose tissues from wild type mouse (BAT UCP1 +/+) and UCP1 KO mouse (BAT UCP1 -/-) were used as positive and negative controls respectively. Data are shown as chemiluminescence chromatograms of anti-UCP1 antibody signals (secondary probe chemiluminescence) and total protein chemiluminescence from four different human donors. Fitted specific UCP1 peak is highlighted in green.

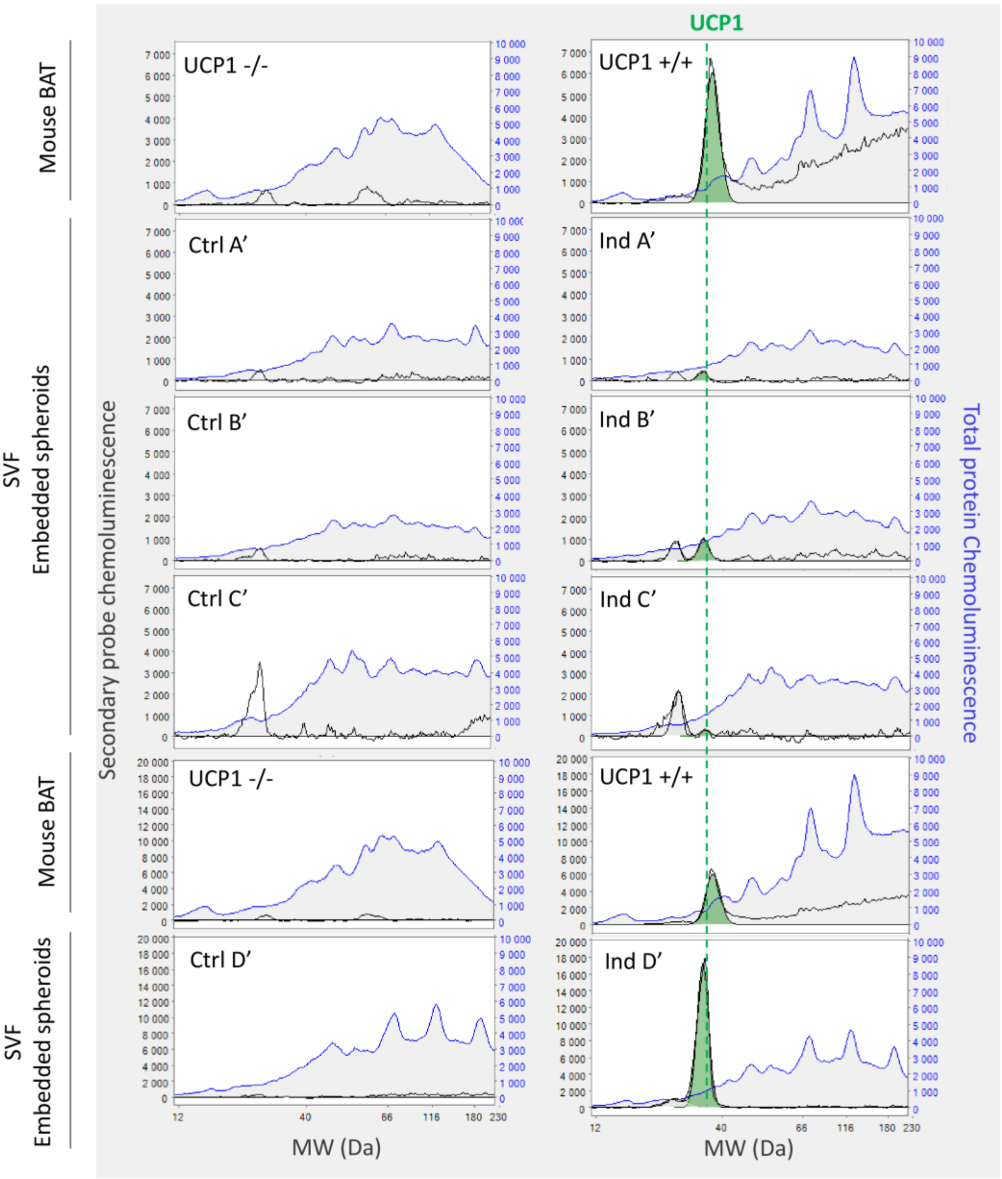

Figure S5. Characterization of UCP1 expression in SVF organoids by proteinSimple capillary electrophoresis immunoassay under control (Ctrl) or induction condition (Ind). Brown adipose tissues from wild type mouse (BAT UCP1 +/+) and UCP1 KO mouse (BAT UCP1 -/-) were used as positive and negative controls respectively. Data are shown as chemiluminescence chromatograms of anti-UCP1 antibody signals (secondary probe chemiluminescence) and total protein chemiluminescence from four different human donors. Fitted specific UCP1 peak is highlighted in green.

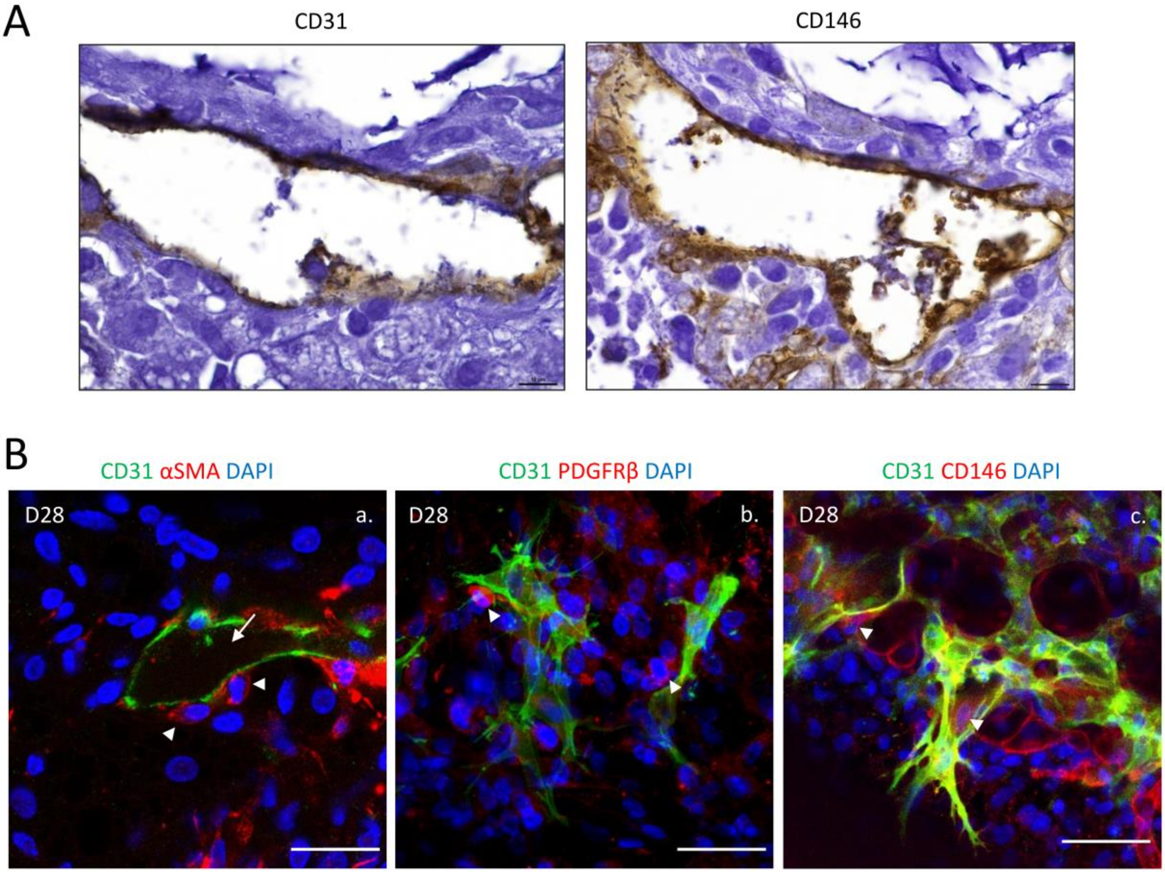

Figure S6: Characterization of vascular lumen formation and perivascular cells within P0-SVF beige adipose tissue organoids. A) Specific anti human CD31 (left) and CD146 (right) were used to characterize the blind developing vascular channels on serial 3 µm sections. The brown positive labelling highlights the positivity of cells delimiting the vascular lumen. Scale bar: 10µm B) Expression of perivascular cells markers was analyzed by immunofluorescence within P0-SVF organoids at the end of the differentiation using confocal imaging. Specific antibodies against human endothelial cell marker CD31 (green) was used in combination of α-smooth muscle actin (SMA) (panel a, red), PDGFRβ (panel b, red) or perivascular and endothelial cell marker CD146 (panel c, red) antibodies. DAPI staining highlights cell nuclei. αSMA expressing cell (panel a, white arrowheads) in close association of endothelial cells showing lumen formation (white arrow) was observed. Some PDGFRβ+ cells (panel b, white arrowheads) associated with CD31+ endothelial cells were also found. Some CD146+/CD31- cells (white arrows) were also observed closely associated to CD31+/CD146+ endothelial cell network. Scale bar: 50 µm.

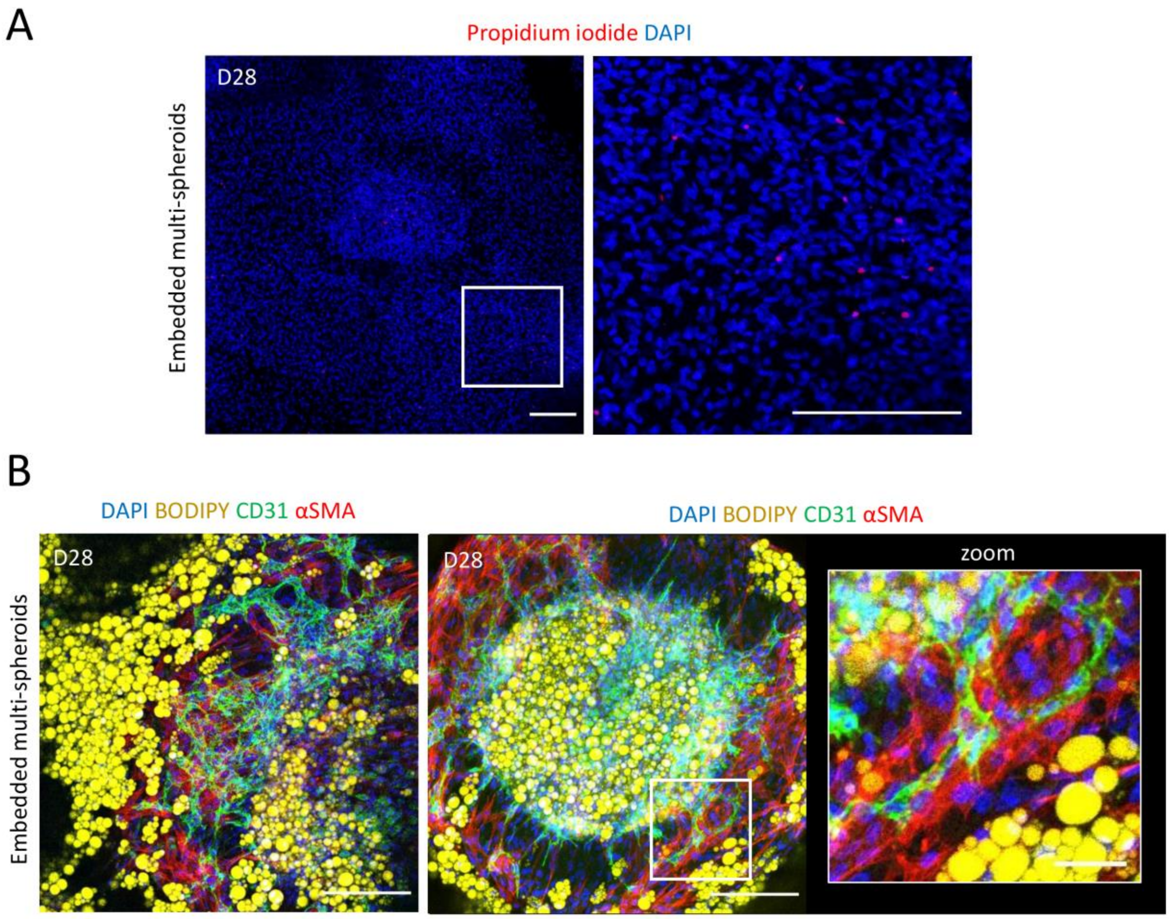

Figure S7. Immunofluorescence characterization of embedded multi-spheroids derived from P0-SVF cells A) Representative confocal images of propidium iodide (Dead cells in red) staining in regards to DAPI (blue) staining within embedded multi-spheroid at day 28. Scale bar: 200 µm. B) Immunofluorescence analysis of P0-SVF embedded multi-spheroids at the end of the differentiation. Specific antibodies against human endothelial cell marker CD31 (green) and α-smooth muscle actin (SMA) (red) were used. Lipids containing cells were revealed by bodipy staining. DAPI staining highlights cell nuclei. Scale bar: 200 µm. Images are two representative areas of embedded multi-spheroids. White squared image shows zoomed area. Scale bar: 50 µm.
